# Field cress genome mapping: Integrating linkage and comparative maps with cytogenetic analysis for rDNA carrying chromosomes

**DOI:** 10.1038/s41598-019-53320-0

**Published:** 2019-11-19

**Authors:** Zeratsion Abera Desta, Bozena Kolano, Zeeshan Shamim, Susan J. Armstrong, Monika Rewers, Elwira Sliwinska, Sandeep Kumar Kushwaha, Isobel A. P. Parkin, Rodomiro Ortiz, Dirk-Jan de Koning

**Affiliations:** 1 Department of Plant Breeding, Swedish University of Agricultural Sciences, Sundesvagen 10, Box 101, SE-23053 Alnarp, Sweden; 2 Department of Plant Anatomy and Cytology, University of Silesia, Jagiellonska 28, 40-032 Katowice, Poland; 3 Mirpur University of Science and Technology (MUST), Mirpur AJK, Pakistan; 4 School of Biosciences, University of Birmingham, Birmingham, B 15 2TT United Kingdom; 5 Laboratory of Molecular Biology and Cytometry, Department of Agricultural Biotechnology, UTP University of Science and Technology, Kaliskiego Ave. 7, 85-789 Bydgoszcz, Poland; 6 Agriculture and Agri-Food Canada, 107 Science Place, Saskatoon, SK S7N0X2 Canada; 7 Department of Animal Breeding and Genetics, Swedish University of Agricultural Sciences, Box 7023, SE 75007 Uppsala, Sweden

**Keywords:** Cytogenetics, Flow cytometry, Genetic linkage study, Comparative genomics

## Abstract

Field cress (*Lepidium campestre* L.), despite its potential as a sustainable alternative oilseed plant, has been underutilized, and no prior attempts to characterize the genome at the genetic or molecular cytogenetic level have been conducted. Genetic maps are the foundation for anchoring and orienting annotated genome assemblies and positional cloning of candidate genes. Our principal goal was to construct a genetic map using integrated approaches of genetic, comparative and cytogenetic map analyses. In total, 503 F_2_ interspecific hybrid individuals were genotyped using 7,624 single nucleotide polymorphism markers. Comparative analysis demonstrated that ~57% of the sequenced loci in *L. campestre* were congruent with *Arabidopsis thaliana* (L.) genome and suggested a novel karyotype, which predates the ancestral crucifer karyotype. Aceto-orcein chromosome staining and fluorescence *in situ* hybridization (FISH) analyses confirmed that *L. campestre*, *L. heterophyllum* Benth. and their hybrids had a chromosome number of 2*n* = 2*x* = 16. Flow cytometric analysis revealed that both species possess 2C roughly 0.4 picogram DNA. Integrating linkage and comparative maps with cytogenetic map analyses assigned two linkage groups to their particular chromosomes. Future work could incorporate FISH utilizing *A. thaliana* mapped BAC clones to allow the chromosomes of field cress to be identified reliably.

## Introduction

The genus *Lepidium* consisting of ~231 species is one of the largest of 338 genera in the Brassicaceae (Cruciferae or Mustard) family^1^. Field cress (*Lepidium campestre* L.) is a self-pollinated, diploid (2*n* = 2*x* = 16) biennial plant originating in Europe^2^ and subsequently appearing as an introduced weed in North America. Smith’s pepperwort (*Lepidium heterophyllum* Benth.) is a diploid (2*n* = 2*x* = 16) perennial plant, and has relatively lower winter hardiness than *L. campestre*^3^.

*L. campestre* has potential as an oilseed crop^3,4^, and could provide an alternative intercrop species with members of the Poaceae family (e.g. barley)^5^. Following the harvest of the main crop, field cress can be engaged as a catch crop during the off-season to prevent soil nutrient leaching^6^, in turn this alleviates underground environmental pollution. Furthermore, field cress could reduce fertilizer dependency (e.g. nitrogen fertilizer), which maintains the soil quality, thereby mitigating the current and future challenges of climate change. These foundational features of the species suggest the possibilities that could arise from domestication of field cress which has yet been underutilized and has been the focus of limited research.

There have been substantial advances in the ability to generate draft genome assemblies for many crop species, as well as in dense genotyping platforms to underpin whole-genome prediction^7^. However, the importance of genetic mapping and molecular cytogenetics remain undiminished, both in supporting these technologies, and in addressing fundamental biological questions related to genome and karyotype evolution. To date, neither a linkage map nor molecular cytogenetic analysis has ever been developed for *L. campestre*. Indeed, cytological investigations of *Lepidium* species have been mainly restricted to chromosome counts^1,8^. *Lepidium* like many of the genera in the Brassicaceae family (e.g., *Arabidopsis* and *Cardamine*)^9,10^ has small and poorly differentiated chromosomes that complicate karyotyping efforts. Hence, additional landmarks are required to identify chromosome pairs. The markers most often used are the 5S and 45S rDNA sequences due to their abundance in the genome and their relatively conserved nature^11,12^.

Meiotic recombination, the reshuffling of genes derived from ancestral gametes during meiosis, allows segregation of parental alleles to be detected in zygote progeny, and hence the inference of order and distance of genes to generate a genetic linkage map that represents a model of the physical chromosomes. Thus, the more meiotic recombination events are captured, the better the resolution of the linkage map^13^. Successful plant improvement strategies predominantly rely on the distribution of crossovers between homologous chromosomes^14,15^. Linkage maps are further employed to detect quantitative trait loci (QTL), to anchor and orient annotated genome assemblies, and to locate candidate genes in fine mapping.

The availability of reference sequences in model plants – such as thale cress (*Arabidopsis thaliana* L.) – sheds contemporary light on the underlying mechanisms of genome architecture evolution. Anchoring linkage maps to the *A. thaliana* genome uncovered highly conserved genomic regions in *Brassica* species^16,17^, indicating that a linkage map could serve as a supplementary toolkit in comparative genomics to translate genomic information between related taxa. Here we analysed with 7,624 single nucleotide polymorphism (SNP) markers the genomes of 501 F_2_ interspecific hybrid individuals resulting in a map with eight linkage groups (LGs). Integrating the SNP loci of field cress into the *Arabidopsis* genome revealed a high proportion (~57%) of sequenced loci were found in regions of conserved synteny between species. We further intertwined the linkage and comparative maps with cytogenetic map, and the cooperative actions of these techniques posited the allocation of LGs into their chromosomes.

Towards the plant domestication of a potentially valuable oilseed plant, we describe herein the aims of (i) developing a linkage map and assigning LGs to their chromosomes in *L. campestre* using integrating techniques of genetic, comparative, and cytogenetic map analyses; (ii) estimating the genome size of *L. campestre* and *L*. *heterophyllum*; (iii) conducting molecular cytogenetic analysis in *L. campestre*, *L*. *heterophyllum*, and their hybrid individuals; and (iv) identifying the ancestral genomic block (GB) structure for *L. campestre*.

## Results and Discussion

### Genome size estimation

The 2C DNA content of the 54 *L. campestre* accessions ranged from 0.394 pg/2C to 0.439 pg/2C, which corresponded to 385 megabase pairs (Mb)/2C and 429 Mb/2C, respectively (Supplementary Table 1, Supplementary Fig. 1). The mean genome sizes of *L. campestre* and *L*. *heterophyllum* were 0.416 pg/2C (407 Mb/2C) and 0.411 pg/2C (402 Mb/2C), respectively. For *L*. *heterophyllum*, this is the first report of genome size estimation. Despite differences in their growth habits, there was no significant difference in mean DNA content between studied species ([ANOVA] *F* = 1.918, degree of freedom [df] = 1, *P* = 0.172). According to the proposed categorization^18^, *L. campestre* and *L. heterophyllum* possess very small genomes (<2.8 pg/2C). A previous report for *L. campestre* genome size (0.7 pg/2C)^19^ and other prior estimates for higher ploidy *Lepidium* species – ranging between 0.66 and 2.08 pg/2C^20^ – were higher than the estimate established here.

### Chromosome counting and fluorescence *in situ* hybridization (FISH) analysis

**Figure 1:**
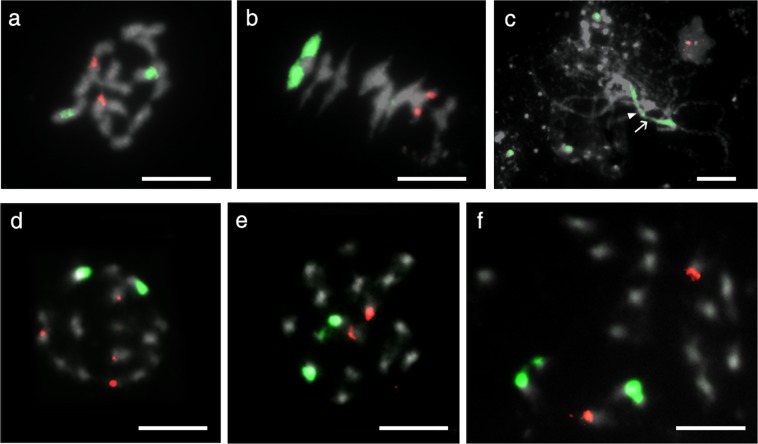
Chromosomal localization of 45S rDNA loci (green fluorescence) and 5S rDNA loci (red fluorescence) in *Lepidium* chromosomes. (**a**) Somatic metaphase. (**b,c**) meiotic chromosomes of *L. campestre* accession coded C54_1 (Supplementary Table 2). The arrowhead and arrow in (**c**) identify the centromere and secondary construction, respectively of a complete chromosome 1 bivalent in an incomplete pachytene cell. (**d,e**) Metaphase plate of *L. heterophyllum* accessions coded as C66_4 and BP1_1, respectively (Supplementary Table 2). (**f**) Somatic metaphase of a hybrid from *L. campestre* and *L. heterophyllum* coded as Hy56_1. Bars represent 5 µm.

**Figure 2:**
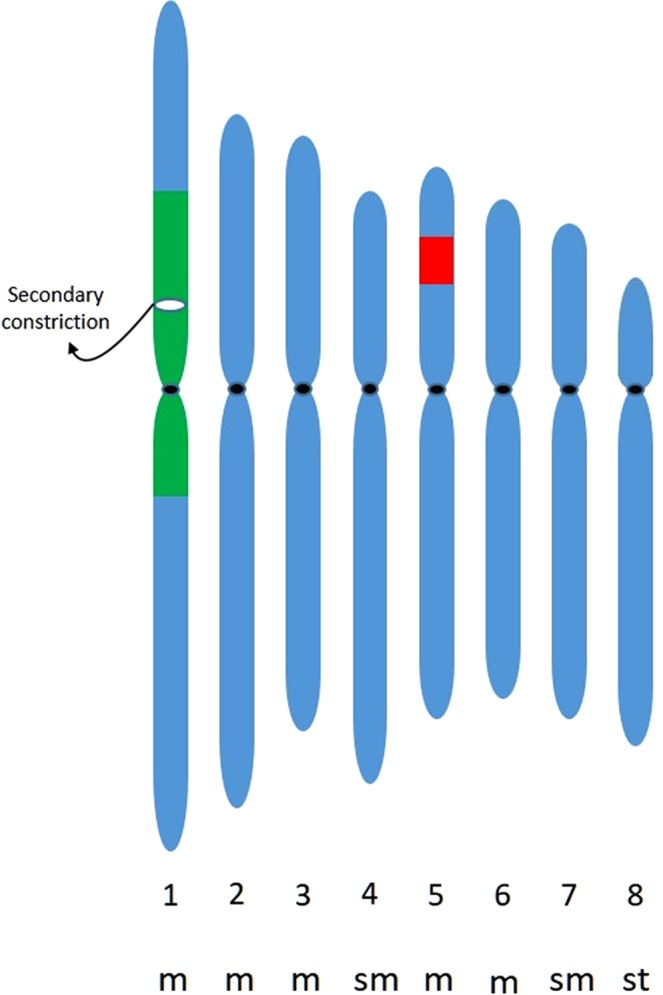
Haploid idiogram representing *Lepidium campestre*. The location of the 45S rDNA (green) and the 5S rDNA (red) are indicated on chromosome 1 and 5, respectively. A black line represents the centromeres, whereas a white line symbolizes the secondary constriction. Abbreviations: m: metacentric chromosome; sm: sub-metacentric chromosome; st: sub-telocentric chromosome.

In addition to chromosome counting in *L. campestre* and *L. heterophyllum* with a standard acetoorcein chromosome staining method, we performed FISH analysis, and both species including their hybrids had 2*n* = 2*x* = 16 chromosomes (Fig. 1a,d–f), in accordance with the published indexes (http://ccdb.tau.ac.il/Angiosperms/Brassicaceae/Lepidium/; http://www.tropicos.org/Project/IPCN). Their karyotypes contained mostly metacentric and sub-metacentric chromosomes, which showed a gradual decrease in their length, except one pair of homologues, which was remarkably longer than the others (Figs 1a,d, 2).

This is the first report of rDNA localization in chromosomes of *L. campestre*, *L. heterophyllum* as well as their hybrids. rDNA sequences can be localized by FISH and can provide cytogenetic markers that are useful for the identification of individual chromosomes^11,21^. Similar to previously described *L. africanum* (2*n* = 2*x* = 16) and many other angiosperms^22,23^, in all analysed *Lepidium* samples (Fig. 1a,d–f), we found that 5S and 45S rDNA loci were localized on different chromosomes. Two hybridization signals for 45S rDNA were observed on mitotic metaphase chromosomes of *L. campestre* and *L. heterophyllum* (Fig. 1a,d,e). In many angiosperms, 45S rDNA loci were most often found in sub-terminal regions of the short arm of the chromosomes^23^. Such localization of 45S rDNA loci was also reported in other *Lepidium* species^22^; however, *L. campestre* and *L. heterophyllum* revealed 45S rDNA loci, both on the short and long arm of the same chromosome, in a more proximal location. To delineate the chromosomal localization of the rDNA, mitotic pro-metaphase chromosomes and meiotic (pachytene and metaphase) chromosomes were probed using FISH. The hybridization signals of 45S rDNA seem to cover most of the short arm, but also span to the proximal part of the long arm in the longest chromosome of *L. campestre* as seen at the pachytene stage, when the chromosomes are paired up and most extended (Figs 1a–c and 2).

The proximal localization of 5S rDNA loci was frequently reported in karyotypes of angiosperms with small chromosomes^23^. As in previous research of other *Lepidium* species^22^, our analysis revealed the 5S rDNA locus in the interstitial position of the short arm of the sub-metacentric chromosomes (Figs 1a,b and 2). In all analysed field cress accessions, only one 5S rDNA locus was detected, whereas *L. heterophyllum* showed intraspecific polymorphism in 5S rDNA locus number, depending on the accession type, one pair (sample code BP1_1) or two pairs (sample code C66_4) of the loci were recorded (Fig. 1a,d,e; Supplementary Table 2). Such a phenomenon was described in many plant genera^11,21^. In addition to the epigenetic nature of the specific locus (5S rDNA)^24^, various mechanisms have been postulated to account for this state such as a transposon-mediated transposition event or chromosome rearrangements caused by homologous or a non-homologous unequal crossing-over and gene conversion^25–27^. Taken together, these results may reflect the high levels of micro-evolutionary or epigenetic changes within these taxa. Furthermore, in a hybrid individual (coded as Hy56_1) (Supplementary Table 2) one pair of 45S rDNA and one pair of 5S rDNA loci were detected (Fig. 1f). Interestingly, the karyotypes of all analysed accessions of *Lepidium* – in both species as well as their hybrid – were similar to each other.

### Genetic linkage map construction

**Figure 3:**
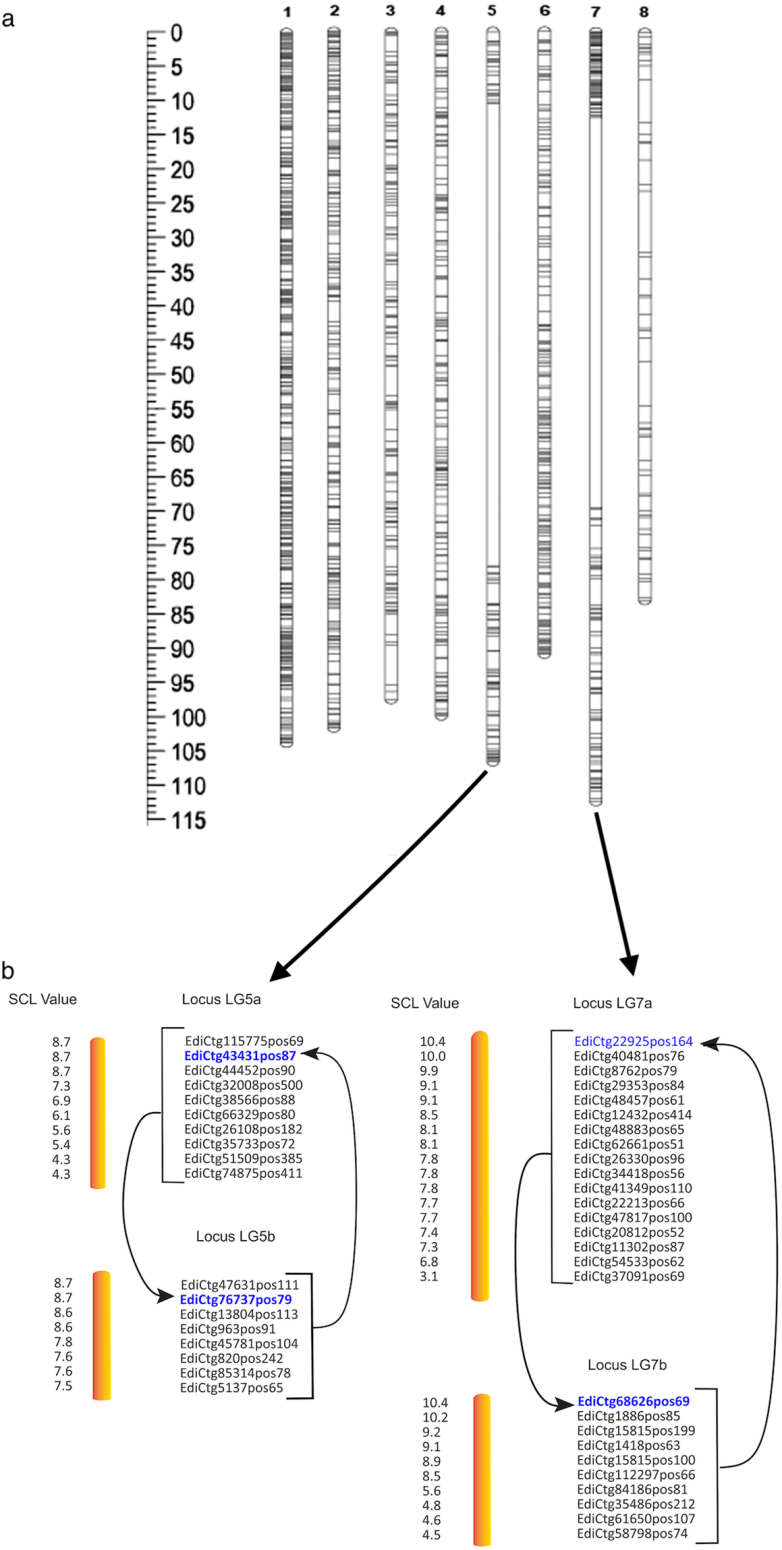
Genetic linkage map constructed for field cress. (**a**) The expected eight linkage groups (LGs) constructed using 1,517 SNP markers. The left side ruler is highlighted to indicate the distance intervals between adjacent loci. (**b**) The strong cross-link (SCL) values used to indicate the loop interplaying routes of loci between the potentially fragmented LG5 and LG7 related to their sub-LGs (Sub-LG5a, sub-LG5b, and sub-LG7a, sub-LG7b, respectively). The subdivision of LGs existed under logarithms of the odds (LOD) threshold values ≥ 9 and ≥11 for LG5 and LG7, respectively. The locus highlighted in blue is an entry to a group of loci from the other sub-divided LG.

In total, 503 F_2_ interspecific hybrid individuals derived from two F_1_ parents (see methods section) were genotyped using 7,624 SNP markers. Segregating SNP loci common to both sub-populations were analysed, and these were combined with the uniquely segregating SNPs from each sub-population. In a combined linkage analysis, 2,016 of the 7,624 SNP markers were scored in 487 individuals. After quality control (see methods section for details), 1,517 SNPs were employed in 482 F_2_ individuals to generate a genetic linkage map for *L. campestre* (Fig. 3a; Supplementary Fig. 3). In the final mapping, 1,401 segregating SNP loci were common to both sub-populations, whereas 116 SNP loci were unique to one or other sub-population (Table 1).

**Table 1:** Summarized distribution of marker loci, map length, and segregation distortion in each linkage group (LG) of field cress

LG	Number of marker locus			Map length (*cM*)	Average distance (*cM*)	Max. gap^c^ (*cM*)	SNP/cM	Sg.D^d^
	Com.^a^	Uniq.^b^	Total					
1	429	5	434	76.35	0.18	1.36	5.7	—
2	217	—	217	75.03	0.69	2.23	3.0	45
3	136	16	152	60.06	0.40	3.60	2.5	85
4	141	42	183	73.99	0.40	1.80	2.5	70
5	85	0	85	93.36	0.39	59.18	1.0	—
6	161	52	213	47.38	0.22	1.25	4.5	181
7	172	1	173	115.90	0.67	58.84	1.5	102
8	60	—	60	24.00	0.40	2.56	2.5	—
Total	1,401	116	1,517	566.07	—	—	—	483
Mean	175.13	14.50	189.63	70.76	0.42	—	—	—

^a^SNP locus common to both sub-populations, ^b^SNP locus unique to one or other sub-population, ^c^maximum gap, ^d^segregation distortion.

The distribution of SNP loci varied in terms of number, density, and distance across LGs. The number of SNPs per LG ranged from the largest LG1 (434) to the smallest LG8 (60) (Table 1). The eight LGs (Fig. 1a, Supplementary Fig. 2) spanned 566.07 centimorgans (cM), with individual LG lengths of 24 cM (LG8) to 115.90 cM (LG7), and a mean density of three SNPs per cM (one SNP per 0.35 cM). The average distance between adjacent SNP loci ranged from 0.18 cM in LG1 to 0.69 cM in LG2, with a mean of 0.42 cM (Table 1). At least one chiasma per LG were captured except for LG8, as this was smaller than 50 cM. For this LG, we are presumably missing some markers because for all of the metaphase I bivalents we have always seen at least one crossover.

### Segregation distortion

A total of 483 (~32%) mapped SNP loci deviated significantly (linkage test: *P* < 0.05) from an expected Mendelian segregation ratio of 1:2:1 (Table 1). The highest number of distorted loci were on LG6 (181), followed by LG7 (102), whereas no distorted loci were found on LG1, LG5, and LG8. Skewed segregation of loci is not uncommon in interspecific hybrids, as already well exemplified in crops such as cotton^28,29^ and rice^30^. Notably, the genetic causes of segregation abnormalities remain largely enigmatic. A number of hypotheses have been proposed to explain such events, for example the degree of divergence of the parental lines^31^, or selection during gamete^32^ or zygote formation^33^. Regardless of segregation distortion, the relative order of loci in linkage maps remains unaffected^34^. Indeed, the inclusion of segregation distortions in highly distant crosses has biological relevance to circumvent further differentiation of LGs^35^. Nevertheless, strict checking of marker data is imperative to control for the spurious association of loci while fitting loci with deviant segregation patterns.

### Evaluation of genetic linkage map

**Figure 4:**
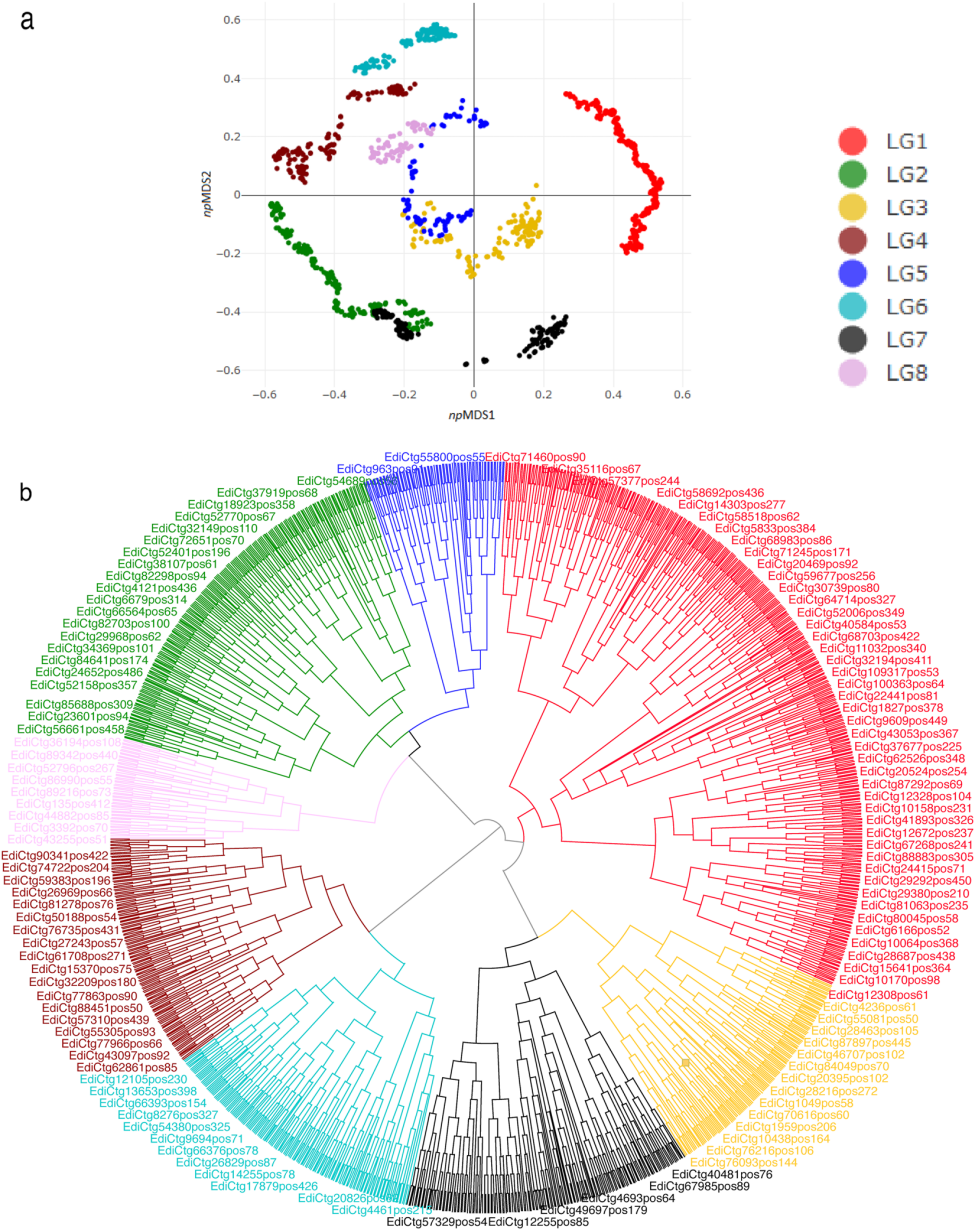
Visualization of loci distribution between and within linkage groups (LGs) of *Lepidium campestre*. (**a**) The 2D *np*MDS plot used to distinguish the structure and distribution of loci between and within LGs. (**b**) Hierarchal cladogram clustering employed to visualize loci positions between and within LGs.

To evaluate the position and distribution of loci within and between LGs, we used various visualization plots. The presence of singletons not only creates erroneous artefacts, but can also merges two unrelated LGs together^35^. When marker loci are separated with a maximum gap of ~>40 cM in the same homologous chromosomes, these markers could actually be unlinked and form different chromosomes^36^. In light of this, we refined the clustering of loci on the basis of patterns apparent in 2D non-parametric multi-dimensional scaling (*np*MDS) ordination plots (Fig. 4a), and hierarchical clustering dendrograms (Fig. 4b) to potentially resolve spurious linkages. In agreement with linkage mapping, the *np*MDS identified the same pattern and distribution of loci within and between LGs, while clustering dendrograms highlighted the distinction of loci between LGs.

To gain additional insight, we computed significant strong cross link (SCL) values (LOD threshold value ~>3 to verify LGs in genetic mapping^35,37^. Based on these values, Fig. 3b reveals the interplay between the sub-groups of the potentially sub-divided LGs (LG5 and LG7) with persistent routes found between them, reflecting that the subdivided LGs were the fusion of their original LGs designation. To scrutinize this further, we explored the relative map position of loci using higher and then lower stringency LOD threshold values^35^, although this approach is less reliable. In this context, we noted similar loci orders (Supplementary Table 3) between the highly stringent (LOD threshold values ≥ 9 and ≥11 for LG5 and LG7, respectively) and the less stringent LOD threshold values (LOD threshold values < 9 for LG5, <11 for LG7), lending additional support that the resulting fragments within these LGs (Fig. 1b) should not be the split into independent LGs. Moreover, the above evidence was corroborated with molecular cytogenetic studies (see the results of the cytogenetic section). Given the similarity karyotype analyses across all samples, we reasoned that the resulting gaps within both LG5 and LG7 possibly stemmed from ancestral rearrangements of chromosomes in both *Lepidium* species (*L*. *campestre* and *L*. *heterophyllum*), and have existed after their evolutionary divergence of the two species.

### Comparative map analysis

**Figure 5:**
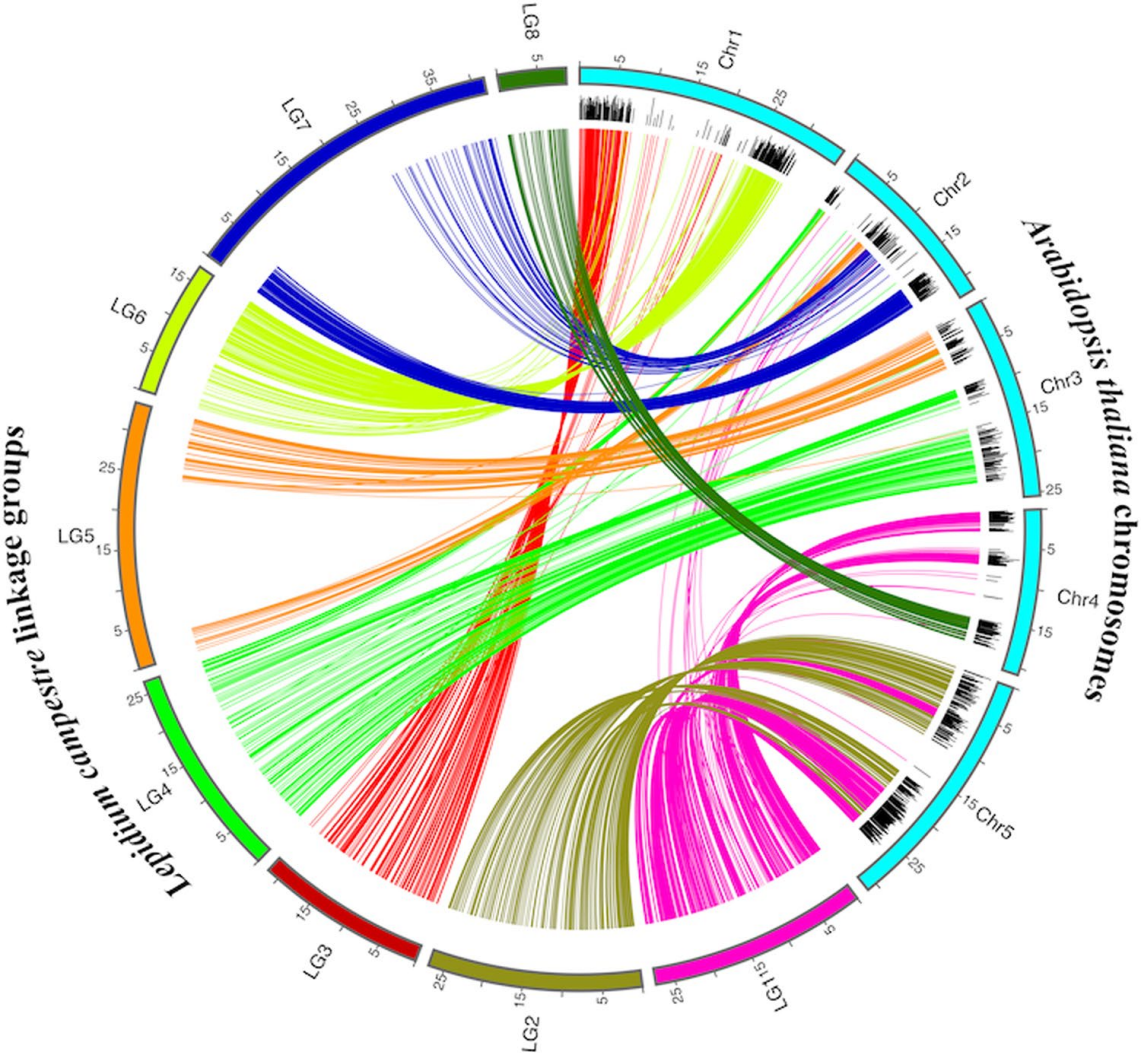
Graphical representation of sequence similarity that reveals the conserved syntenic regions between linkage groups (LGs) of field cress and chromosomes of *Arabidopsis thaliana*. The black bars show the level of similarity of SNP loci against the *Arabidopsis* genome.

The local alignment search tool nucleotide (BLASTN) search using *L. campestre* SNP sequences showed ~83% (1,254) of the 1,517 SNP loci shared sequence similarity with the *A. thaliana* genome (Table 2; Supplementary Table 4). However, the presence of sequence similarity or BLAST hits between species does not necessarily reflect their syntenic relationships^38^. In our comparative map analysis, ~69% (866) of the 1,254 similar sequence loci (or ~57% of the 1,517 polymorphic sequence loci) were congruent with the *Arabidopsis* genome (Fig. 5; Table 2). The relative reduction in homologous loci (from ~69% to ~57%) probably due to the occurrence of translocation, fusion or fission of genes after the evolutionary divergence of these two species^39^. However, the 866 homologous loci identified highly conserved regions of the genomes after the evolutionary divergence of the two species. The number of conserved syntenic loci per LG varied between 36 (LG5) and 280 (LG1) (Table 2).

**Table 2:** The 1,517 sequence loci in eight linkage groups (LGs) of field cress related to indels, duplications, collinearity, and homology with *Arabidopsis* genome

LG^a^	Marker locus	Blast hit	Ins^b^	Del^c^	Dup^d^	Homol. loci^e^	Loci homologous to *A. thaliana* chromosomes				
							Chr1^f^	Chr2	Chr3	Chr4	Chr5
LG1	434	356	6	13	22	280	—	2	—	109	169
LG2	217	179	5	5	4	107	—	—	—	—	107
LG3	152	135	2	6	6	96	96	—	—	—	—
LG4	183	144	4	1	2	74	—	14	60	—	—
LG5	85	70	1	1	—	36	—	9	27	—	—
LG6	213	168	4	4	4	127	127	—	—	—	—
LG7	173	146	1	3	6	104	—	104	—	—	—
LG8	60	56	3	1	2	42	—	—	—	42	—
Total	1,517	1,254	26	34	46	866	233	129	87	151	276

^a^linkage group, ^b^insertions, ^c^deletions, ^d^duplications, ^e^homologous loci, ^f^*A. thaliana* chromosome.

Comparative mapping is not only used to elucidate QTL bearing regions, but also to engage in positional cloning of genes^40,41^. The characterization of flowering time in the *Arabidopsis* genome is one of the notable examples in translating genomics between related species. The *Flowering Locus C* *(FLC*) gene has been found to underlie a major domestication QTL, which inhibits flowering in plants^42,43^. The orthologous analyses of *FLC* genes unveiled common regions between *Brassica* species and *Arabidopsis* genome^44,45^. Since ancestrally conserved regions typically harbour functional genes, mapping genomic regions and exploiting known synteny relationships, could be a keystone to accelerate the future domestication and improvement of field cress.

Next, we quantified 60 insertion − deletions (indels) that ranged from two on LG5 to 18 on LG1 (Table 2, Supplementary Table 4). We speculate that the presence of only a few indels might be either because of losses in diversity (e.g., small effective population size)^46^ or because of the short evolutionary history of divergence accompanied by slow evolution rates of indel polymorphisms^47^. To this end, however, we note further efforts are needed for gaining an in-depth understanding of the underlying genomic plasticity that result in indel variation.

The comparative study between field cress and *Arabidopsis* yielded 46 nuclear duplications in seven of the eight LGs (Table 2, Supplementary Table 5). Lui and Adam^48^ found the expression of *SHORT SUSPENSOR* (SSP) gene was retained following duplication, suggesting neofunctionalization (new functions) in genes controlling brassinosteroid signal transduction of *Brassica* species. The *Conserved Telomere Maintenance Component 1* (*CTC1*) was one of the paradigmatic duplications in *L. campestre* found in LG1 (Supplementary Table 4); however, further functional validation of the *CTC1* gene is crucial. In higher plants, the interaction of *CTC1* with SRN1 led to a new function that integrates and maintains the telomere regions of the chromosome^49^. Collectively, these findings indicate that exploring the conserved syntenywith *A. thaliana* can provide biological insights to unlock genes that underlie traits of interest in field cress.

### Defining the ancestral genomic block structure for field cress

**Figure 6:**
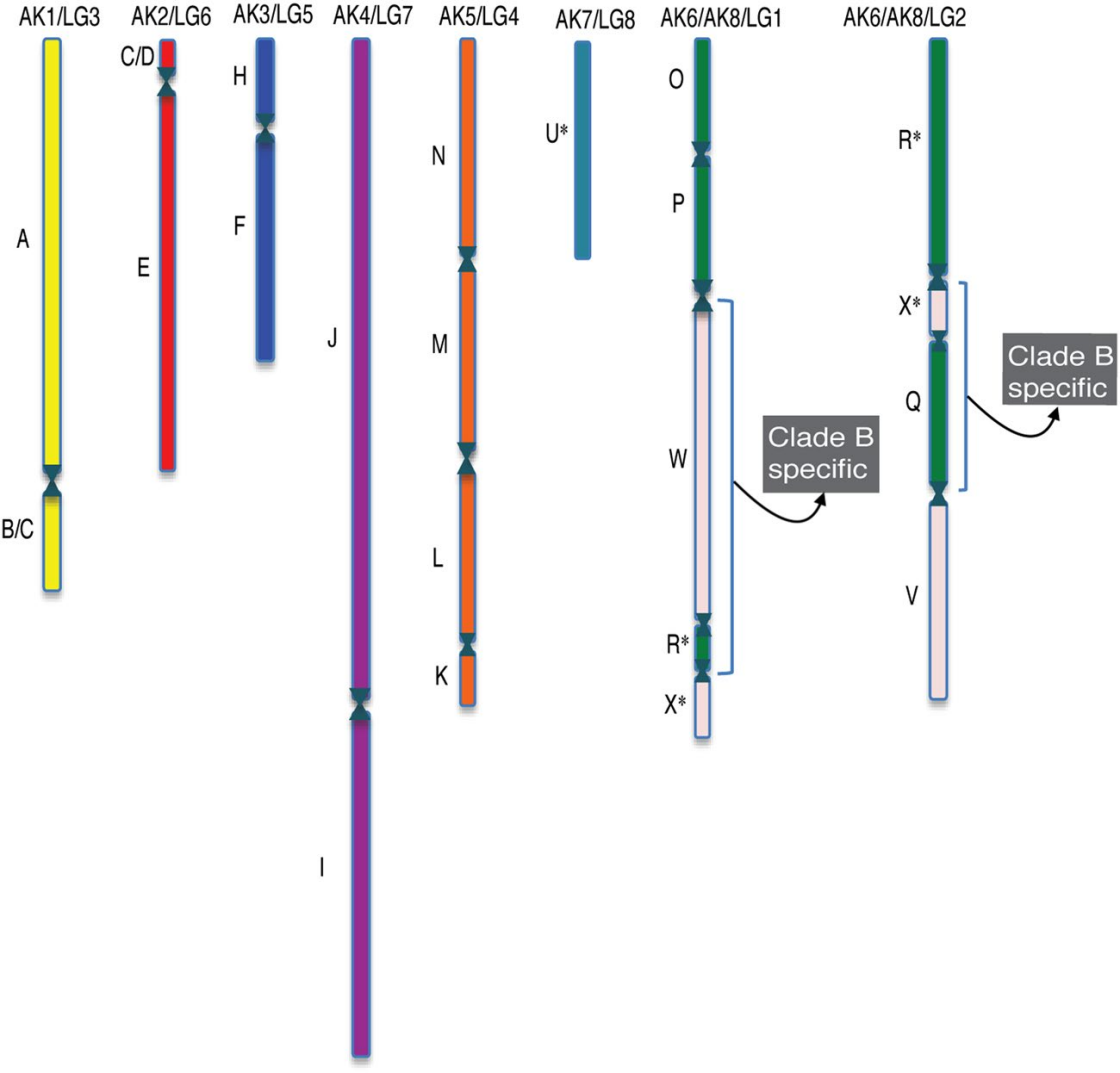
Graphical representation of the ancestral genomic block (GB) structure (A–X) of the field cress genome. The linkage groups (LGs) are named according to their correspondence to each of the chromosomes of the ancestral crucifer karyotype (ACK), ACK1–8. Two of the *L. campestre* LGs are rearranged compared to the ACK and each carries a GB association (R–W and Q–X) distinctive of the karyotype of related clade B species.

The study of genome evolution within the Brassicaceae has been facilitated by the definition of 24 ancestral genomic blocks (GBs), A- X, that are identified based on conserved gene content and order across all species of the family studied to date^50,51^. Utilizing the conserved synteny mapped between *A*. *thaliana* and *L. campestre* genomes, it was possible to identify 21 of the 24 GBs (Fig. 6, Supplementary Table 6), three of the blocks (G, S and T) were not found, which can be explained by their consistent peri-centromeric location that often leads to a paucity of useful polymorphic markers. *L. campestre* in most recent studies has been placed at the base of clade A (or lineage I) of the Brassicaceae^52^ and would be expected to have evolved from the previously characterized ancestral crucifer karyotype (ACK). Indeed, six of the *L. campestre* linkage groups share a common GB structure with six of the ACK chromosomes (Fig. 6). However, LG1 and LG2 of *L. campestre* are rearranged from two ACK chromosomes (AK6 and AK8) and most interestingly show ancestral GB associations (R-W and Q-X; Fig. 6) that have not been documented in 18 previously studied clade A species and were thought to be indicative of species from clade B^51^. The karyotype of field cress effectively links clade A and B and the basal position of the species within clade A suggests that this novel karyotype pre-dates the ACK.

### Assigning LGs to their chromosomes

A key challenge in genetic mapping is the reliable alignment of LGs to their chromosomes. This becomes even more problematic when genotyping is done in the absence of a reference genome, which is the case for *L. campestre* in this study. Thus, additional techniques such as cytogenetic and physical map analyses could reliably  orient LGs to their chromosomes. Given the cytogenetic analysis, the present findings are helpful for an initial overview to assign LGs to their chromosomes. To estimate the LG map based on diakinesis or metaphase I cells, we inferred the number of crossovers and found a mean crossover number of 13, equivalent to 650 cM (13 multiplied by 50 cM).

The relatively small reduction (13%) in size between genetic map (566.07 cM) (Table 1) and cytogenetic map (650 cM) (Fig. 5) is not unexpected, as linkage is highly sensitive to various factors such as accuracy of genotype scoring, representation of recombinants in the population, density of markers, and other constraints of mapping estimations^53^. This is exemplified by LG8 at 24 cM, which could result from a shortage of polymorphic markers. A minimum of one crossover would be expected because of the requirement that at least one obligate crossover (a size of 50 cM) was needed for normal segregation and perfect fertility^54^. Thus, the LG map is likely to be expanded as more markers become available.

Extrapolating chromosome 1 from the defined karyotype (Fig. 2) suggests it is highly likely related to LG7 for the following reasons. First, chromosome 2 in *A. thaliana* has a large site for 45S rDNA, and it is evident that chromosome 2 and LG7 share substantial conserved syntenic regions (Fig. 5, Table 2). Second, there is a large gap on LG7, which could be due to the presence of a large 45S rDNA site (Fig. 2). And third, an estimate of crossovers from chromosome 1 indicates that it is at least twice 100 cM in size (on average two chiasmata), which is almost  commensurate with the longest LG (LG7 = 115.90 cM; Table 1). Although it was the longest among the LGs or chromosomes, the genetic map was unable to capture any crossovers in the heterochromatic regions of chromosomes, which may be the site of 45S rDNA, known as ‘coldspots’^55^. Considering the point that there appears to be discontinuous LGs  for chromosomes 1 and 5, this may be due to an early reorganization of these chromosomes, prior to separation of the chromosomes  into field cress and *L*. *heterophyllum*, and predating the organisation of *Arabidopsis*.

In similar manner, we explored chromosome 5 (of the defined karyotype in Fig. 2), which could possibly be aligned to LG2. The major locus of 5S rDNA in *A. thaliana* karyotype is placed on chromosome 5, and in turn, LG2 is congruent with chromosome 5 (Fig. 5, Table 2). Moreover, we found two crossovers between the long arms of chromosome 5 in metaphase I bivalents, suggesting an estimated size of 100 cM, which is comparable to the length of LG2 (Table 1). Though our analysis suffers from ascertainment bias owing to the lack of reference genome, we have assigned two LGs to their particular chromosomes. To ensure the alignment of LGs to their chromosomes reliably (e.g. as previously carried out in *Brassica oleoracea*^53^) future work could include the integration of mapped BAC clones in *Arabidopsis* or other markers developed for *L*. *campestre* as FISH probes. These analyses may also provide in developing specific chromosome markers for *L*. *campestre*.

## Conclusion

In summary, our results provide the first glimpse of the genome of field cress and have posited on how to integrate multiple techniques – genetic map to comparative and molecular cytogenetic maps – elucidating the resources to effectively assign two LGs to their chromosomes. Each technique individually cannot be guaranteed to identify chromosomes, instead their joint contributions not only correlate LGs to their chromosomes, but also assist in verifying LG contiguity when large gaps are present in the genetic map. Intriguingly, the detection of huge heterochromatin regions (Fig. 2) was  supported with overlapping evidence in both FISH and linkage map analyses.

Using cytogenetic analysis, strikingly similar regions of the chromosome inert for crossovers were detected in both species (field cress and *L*. *heterophyllum*) including their hybrid individual, suggesting that these regions may be derived from their progenitor’s chromosomal rearrangements of these two species. However, additional efforts in integrating BAC clones of *Arabidopsis* or other FISH probe markers for field cress genome could assign reliably the fragmented LGs to their chromosomes. Genetic mapping is the indirect representation of marker loci in a LG; in other words, it does not reflect the physical presence of nucleotide bases in a chromosome. Notwithstanding this limitation, by exploiting conserved synteny our results could plausibly contribute to identifying candidate QTL in marker-assisted selection (MAS), genome-wide association studies (GWASs), and genomic selection.

Apart from the integrated genome mapping, here we report for the first time – to our knowledge – FISH analysis in *L. campestre* and *L. heterophyllum* along with their hybrid individual. We have uncovered intraspecific polymorphism in 5S rDNA loci number in *L. heterophyllum*, which could suggest either a relatively high level of microevolutionary changes or epigenetic behaviour of this specific locus (5S rDNA) within this taxon.

Based on comparative genome analysis, anchoring the linkage map of field cress to the *Arabidopsis* genome has revealed several regions of conserved synteny, which will play a substantial role in the discovery of novel genes or alleles that maybe utilized in the domestication and molecular breeding of *L*. *campestre*. Interestingly, field cress (a member of lineage I or Clade A) shares common ancestral genomic block organisation with members of species in the lineage II or Clade B (which includes all *Brassica* species). This finding is a novel compared to all previously studied members of lineage I^52^ and suggests an alternative organisation for the ACK, which is currently the foundation of all Brassicaceae comparative mapping.

## Methods

### Flow cytometry

Samples of young leaves of *Lepidium* and an internal standard (*Raphanus sativus* cv. Saxa, 1.11 pg/2C)^56^ were prepared as previously described^57^, using Galbraith’s buffer^58^, supplemented with propidium iodide (PI; 50 μg/mL) and ribonuclease A (50 μg/mL) for nuclei isolation. The suspension of nuclei was analyzed directly after preparation using a CyFlow SL Green (Partec GmbH, Münster, Germany) flow cytometer. For each sample, nuclear DNA content in 3000–6000 nuclei was measured, using linear amplification. Analyses were performed on 48 and three samples of *L. campestre* and *L. heterophyllum*, respectively (Supplementary Table 1). Histogram plots were evaluated using FloMax software (Partec GmbH, Münster, Germany). The coefficient of variation (CV) of the G_0_ /G_1_ peak of *Lepidium* species ranged between 3.52 and 7.05%. Nuclear DNA content was calculated using the linear relationship between the ratios of the 2C peak positions of *Lepidium* /*Raphanus* on a histogram of fluorescence intensities. The 2C genome sizes in picograms (pg) were converted to mega base pair (Mb) using the formula 1 pg = 978 Mb^56^. One-way ANOVA was performed to check the significant difference in the genome size between and within the species.

### Chromosome counting

Chromosome counts were made from root-tip meristems of young seedlings. Seeds were placed on wet filter paper (65% humidity) and incubated in the  growth chamber at 23 ± 1 °C in 16 /8 h photoperiod. After radicle protrusion isolated root-tips were pretreated with 0.002 M 8-hydroxyquinoline for 2 h at 16 °C in darkness. Afterwards, the material was fixed in absolute ethanol and glacial acetic acid (3:1, respectively) for 24 h at 4 °C, and then stored in 70% ethanol at 4 °C. Meristems were stained with 1% aqueous aceto-orcein solution for 24 h, and squashed on slides in 45% acetic acid: glycerol solution (9:1, respectively). Chromosomes were counted on at least 50 metaphase plates for each species and were photographed with a CAMEDIA C-7070 digital camera (Olympus) mounted on a BX41 microscope (Olympus).

### Molecular cytogenetic analysis

For cytogenetic analyses, three accessions of *L. campestre*, two accessions of *L. heterophyllum*, and three hybrid samples (Supplementary Table 2) were used. Mitotic chromosomes isolated from the root meristems and tapetal cells as well as meiotic chromosomes at the pachytene and metaphase I stage in pollen mother cells were used for cytogenetic studies.

The seeds were germinated on agar plates, and the seedlings were vernalized at 4 °C for six weeks. We next transferred seedlings to a soil-based compost and grew in a glasshouse on average temperature of 18.5 °C under 16 h light, and 8 h of dark cycles. For chromosome preparation, primary inflorescences were harvested during inflorescence initiation stage. Individual stamens from suitable buds were dissected to get mitotic and early meiotic stages under a stereo microscope by using lacto-propionic orcein stain and a squashing method. The remaining anthers from these buds were fixed in fresh ice-cold fixative (ethanol and acetic acid in the ratio 3:1).

The protocol of chromosome preparations^53^ was carried out as follows. Fixed anthers or buds were washed with citrate buffer (10 mM, pH 4.5) three times for five minutes each before being subjected to enzyme digestion for up to  3 h in a moist chamber at 37 °*C*. The digestion medium comprised of 0.1% (w/v) cellulase, 0.1% (w/v) pectolyase (Sigma) in 10 mM citrate buffer *pH* 4.5, which breaks down the cell walls during the incubation period. After incubation in the enzyme mixture, anthers were squashed using a needle in a drop of 60% acetic acid and kept on the hot block at 45 °*C* for 1 minute. Following this, the material on the slide was fixed with ice cold fresh fixative and poured around the acetic acid drop. The slides are tilted and flushed with cold fixative before air-drying. Thereafter, they are observed with a phase contrast microscope for suitable meiotic and mitotic stages.

For mitotic chromosome preparation from root meristems whole seedlings (approximately 3 cm long) were pretreated with 2 mM 8-hydroxyquinoline for 6 h and fixed in 3:1 ethanol/acetic acid. After several washes in 0.01 M citric acid-sodium citrate buffer (pH 4.8), the excised roots were subjected to enzymatic digestion in a mixture comprising 20% pectinase (Sigma P0690) and 2% cellulose (Onozuka R-10 Serva) for 45 min at 37 °*C*. Meristems were dissected out from root tips, and then squashed in a drop of 45% acetic acid on microscope slide. After freezing, coverslips were removed and the slides were air-dried.

The probe used for detection of 45S rRNA gene loci was a clone pTa71 containing a 9 kb EcoRI fragment of *Triticum aestivum*, consisting of the 18S-5.8S-25S rRNA genes and the non-transcribed intergenic spacer regions (EMBL X07841)^59^, labelled with fluorescein-12-dUTP (Sigma). For detection of 5S rDNA sites, a 500-bp clone pCT4.2 isolated from *A. thaliana*^60^ was labelled with biotin- 16-dUTP (Sigma). Both DNA probes were labelled by nick translation (Roche). FISH was preformed according to the protocols published earlier^21,61^. The selected slides are washed in 2*X* SSC solution for 10 min at room temperature before being subjected to pepsin (0.01 gpepsin in 0.011 MHCL (hydrochloride)) digestion at 37 °C for 90 sec. The slides were then washed with 2 *X* SSC twice for 5 mineach. The slides are fixed in cold 4% paraformaldehyde at room temperature for 10 min under a fume hood and washed with distilled water twice for 1 min each.

Next, slides were dehydrated in ethanol series of 70%, 85% and 100% for 2 min each at room temperature. The slides were left to dry for 30 min at room temperature until all ethanol has evaporated completely. The hybridization mixture consisting of 100 ng of each labelled DNA probe, 50% formamide, 10% dextran sulphate and 2×SSC was denatured for 10 min at 85 °C, then chilled on ice and applied to the chromosome preparation. The slides and hybridization mixture were denatured together at 70 °C for 4 minin an *in situ* Thermal Cycler (ThermoHybaid, Franklin, USA), and allowed to hybridize in a humid chamber at 37 °C for 24–48 h. Stringent washes (71%; 10% formamide in 0.1 *X*SSC at 37 °C for mitotic material and 82%; 50% formamide in 2 *X*SSC at 45 °C for meiotic material)^21,62^ were followed by the immunodetection of biotin using Cy3-conjugated streptavidin (Sigma).

The preparations were mounted in Vectashield (Vector Laboratories, Peterborough, UK) containing 2 μg/ml DAPI. Images were acquired with camera Retiga-2000R Fast1394 mounted on an Olympus Provis epifluorescence microscope, processed uniformly and superimposed using Smart Capture (Digital Scientific) and Adobe Photoshop software. For meiotic preparations the slide was viewed with an Olympus fluorescent microscope connected to an image analysis system, (Digital Scientific). Karyotyping is performed using Smart Capture software (Digital Scientific). Good spreads of mitotic chromosomes were selected for  analysis on the SmartType Karyotyper.

The idiogram for *L. campestre* was constructed (Fig. 2) based on 10 good-quality tapetal mitotic metaphases, which have been hybridized with both 45S and 5 SrDNA, and counter stained with DAPI. The separation and sorting of chromosomes were achieved using Smart Capture software (Digital Scientific). Once all the selected images were karyotyped, the length of each chromosomes pair was measured on image-J and recorded. The centromeric index was calculated as a ratio of short arm length to the total chromosomal length. The idiogram displayed in Fig. 5 was constructed using the relative length and centromeric index. Because the cytogenetic map of all samples of *L. campestre*, *L*. *heterophyllum* along with their hybrids are inseparable; only one representative idiogram in *L. campestre* is presented in this article.

### Mapping population

Crosses between two *L. campestre* (female parents) and *L. heterophyllum* (common pollen donor parent) generated two F_1_ interspecific hybrid progeny. Subsequent selfing of these F_1_ plants produced two half-sib sub-populations consisting of 503 F_2_ interspecific hybrid individuals as a mapping population. The two sub-populations with 246 and 257 individuals were referred to as sub-population 1 and sub-population 2, respectively.

### DNA extraction and SNP genotyping

DNA was harvested from young and fresh leaves of field cress using cetyl trimetheyl ammonium bromide (CTAB) method^63^, which was modified to double extraction and purification techniques. DNA samples of the 503 F_2_ individuals were sent for genotyping using iSelect Illumina Infinium technology to Edinburgh Genomics (https://genomics.ed.ac.uk). SNPs on the custom-made iSelect Illumina array were selected from restricted amplified DNA (RAD) sequences that were obtained earlier on 18 *Lepidium* samples^64^. The FASTA file of all the RAD sequence data can be accessed at: 10.6084/m9.figshare.9786020.v1. Poor quality SNPs were noticed by their cluster separation values (low Norm R and Norm Theta scores). These poor score SNPs were excluded, and eventually 7,426 SNPs were employed to genotype the 503 F_2_ individuals.

### Genetic map construction

SNP loci (1,603) and individuals (13) with ≥5% of missing values were excluded from mapping. SNP loci with extremely skewed segregation (*P* < 0.0001) towards one of the two parents (2,020 loci) were excluded to deliver 4001 loci in 490 individuals. In addition to the identical individuals (six) and odd recombinants (two individuals), similar loci and singletons (odd representation of SNP loci positions in a LG) totalling 2,484 loci were precluded. After pre- and post-map quality inspection, 1,517 of the 7,624 SNP loci and 482 of the 503 individuals were retained in the final genetic linkage map construction (Fig. 3, Table 1, Supplementary Fig. 2). A linkage map was implemented using JoinMap 4.1 version software^37^, relying on a maximum likelihood of mapping algorithm. The independence logarithms of the odds (LOD) parameter ranging from three to 11 were subjected to classify linked markers to their linkage groups (LGs). Multi-point estimation of recombination frequencies was handled using Gibbs sampling procedure. This parameter was amended to 15,000 and 2,000 related to the length of burn-in chain and the chain length per EM (expectation maximization) cycle, respectively.

The simulated annealing parameters were used for map order optimization, and the chain length was adjusted for 30,000, cooling control parameter to 0.0001, the stop number of chains without improvement to 20,000. Thresholds of five recombination frequencies (that is, 0.100, 0.050, 0.030, 0.020, and 0.010) were employed as spatial sampling procedure to prevent the map from reaching the local optimal. The mapping function was computed using Haldane’s mapping function^65^ and linkage map was illustrated in MapChart 2.2 software^66^.

The structure and distribution of marker loci were visualized (Fig. 4) using 2-D non-parametric multi-dimensional scaling (*np*MDS), and hierarchical clustering in R version 3.4.1 software package^67^ while visualization of trees were implemented using Dendroscope 3 (version 3.5.9)^68^. The Manhattan distance using average method was employed in clustering using factoextera package^69^. The dimensional distances in *np*MDS with *iso*MDS package were computed using 1−cor Spearman’s correlation as this treats non-continuous data more vigorously than Pearson’s correlation coefficient^70^.

### Comparative map analysis

To discover putative sequence similarities in the flanking marker loci of *L. campestre*, a basic local alignment search tool nucleotide (BLASTN) search was performed against the coding sequences (CDS) and genomic sequences of *A. thaliana* genome (TAIR; www.arabidopsis.org) (Supplementary Table 4). We used the *s*liding window approach with word length, W = 9 and significant E-value cutoff 1e-05 settings.

The BLASTN results were sorted according to genetic distance (cM) in ascending order within each LG. We wrote a PERL script to identify the gene orientation and order of marker loci as prescribed^38^. Subsequently, neighbouring genes of marker sequences were identified with an extension of 120,000 base pairs in both sides of each query of sequence loci compared to *Arabidopsis* chromosome within clusters of at least three genes (Supplementary Table 4). The syntenic regions between *L. campestre* and *A. thaliana* genome were visualized using Circos-0.69 software package (Fig. 5)^71^. To perform gene duplications of *Lepidium*, initially, self-BLAST to *Lepidium* SNP sequences was carried out, in turn the output was employed as substrate for MCL-edge software^72^ to recognize the duplicate and multi-member gene families (Supplementary Table 5). The synteny data was utilized to identify the ancestral genomic block structure of *L. campestre*. The likely ancestral origin of each locus was determined based on its homology to *A. thaliana* and the position of the corresponding *Arabidopsis* gene within the pre-classified ancestral blocks (Supplementary Table 6)^50^.

## Supplementary information

Combined supplementary files for Figure 1 & 2, and Table 1, 2, & 5

Supplementary Table 3. Comparisons of linkage map positions in the fragmented LG5 and LG7

Supplementary Table 4. BLASTN and conserved synteny of Lepidium campestre in comparison with Arabidopsis genome

Supplementary Table 6. Identifications of ancestral crucifer karyotype (ACK) in Lepidium campestre
