# Supplementary material for: Field cress genome mapping: Integrating linkage and comparative maps with cytogenetic analysis for rDNA carrying chromosomes
Source: Sci Rep. 2019 Nov 19;9:17028. doi: 10.1038/s41598-019-53320-0 (PMC6863836; doi:10.1038/s41598-019-53320-0)
Supplement: Supplementary file 1 — Combined supplementary files for Figure 1 & 2, and Table 1, 2, & 5 [file 41598_2019_53320_MOESM1_ESM.pdf]

Field cress genome mapping: Integrating linkage and comparative maps with cytogenetic analysis for rDNA carrying chromosomes

Zeratsion Abera Desta[1*], Bozena Kolano[2], Zeeshan Shamim[3,4], Susan Armstrong[4], Monika Rewers[5], Elwira Sliwinska[5], Sandeep Kumar Kushwaha[1], Isobel A. P. Parkin[6], Rodomiro Ortiz[1], Dirk-Jan de Koning[7]

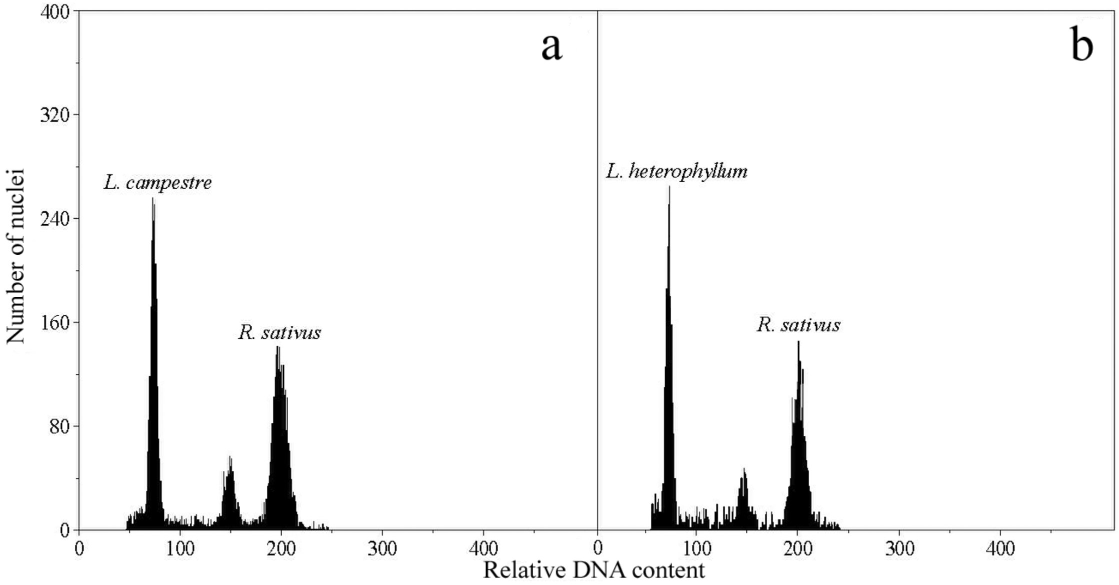

Supplementary Fig 1. Histograms of nuclear DNA content obtained after flow cytometric analysis of the nuclei isolated simultaneously from the leaves of *Raphanus sativus* cv. Saxa (internal standard) and *Lepidium*. a, *Lepidium campestre*. b, *Lepidium heterophyllum*.

Linkage group1

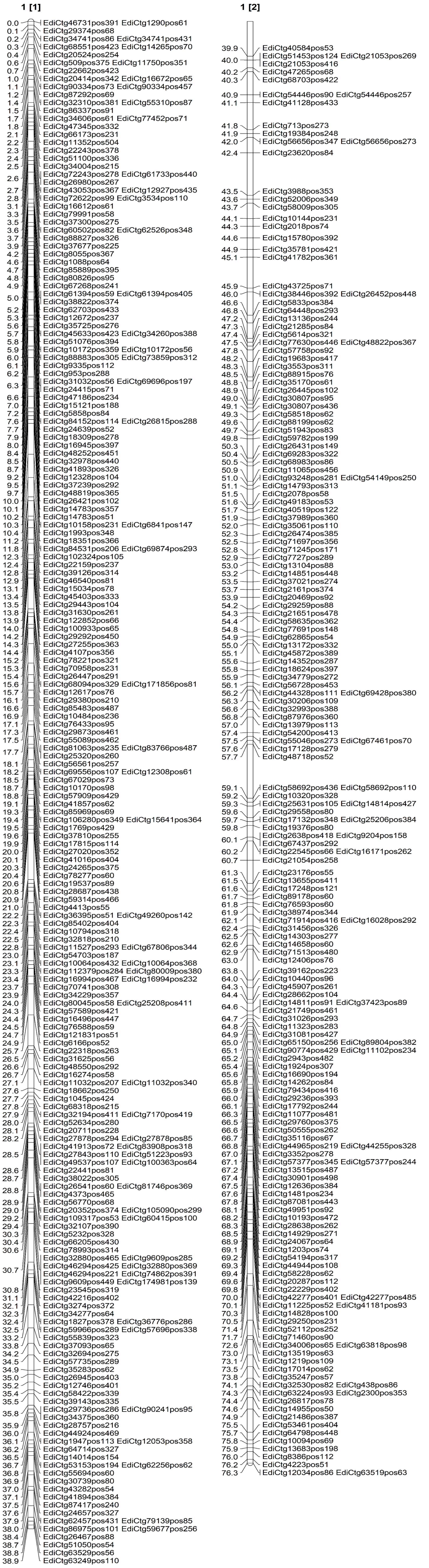

Linkage group 2

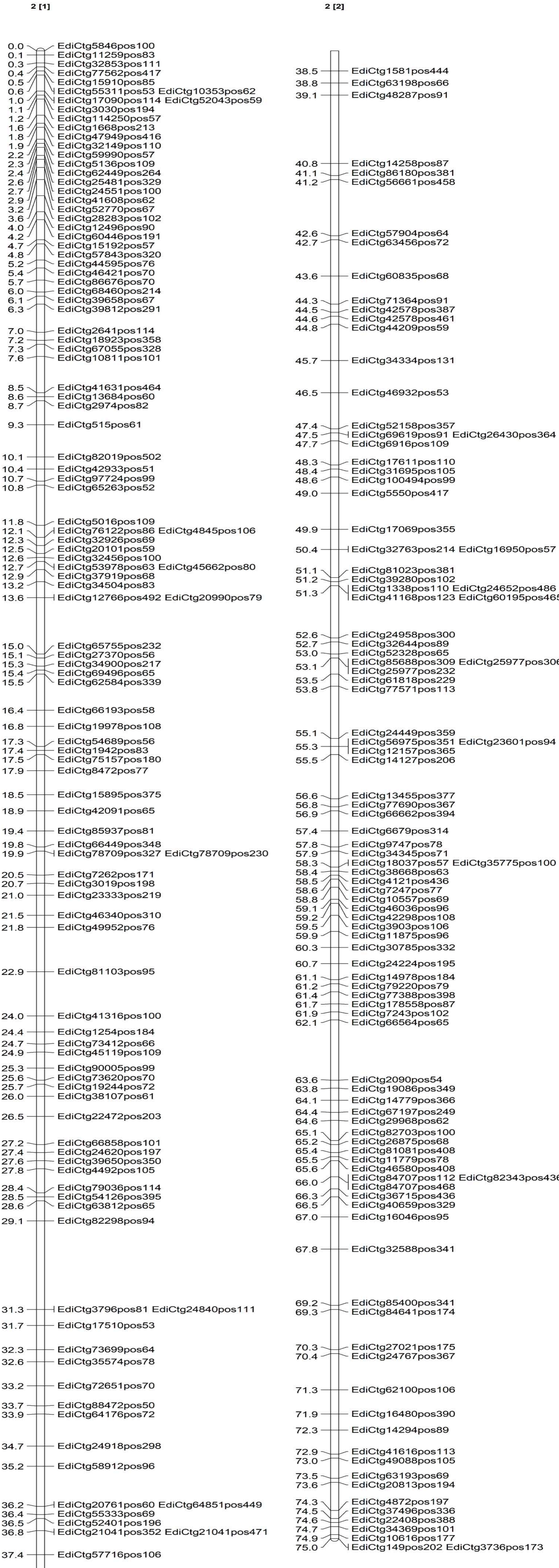

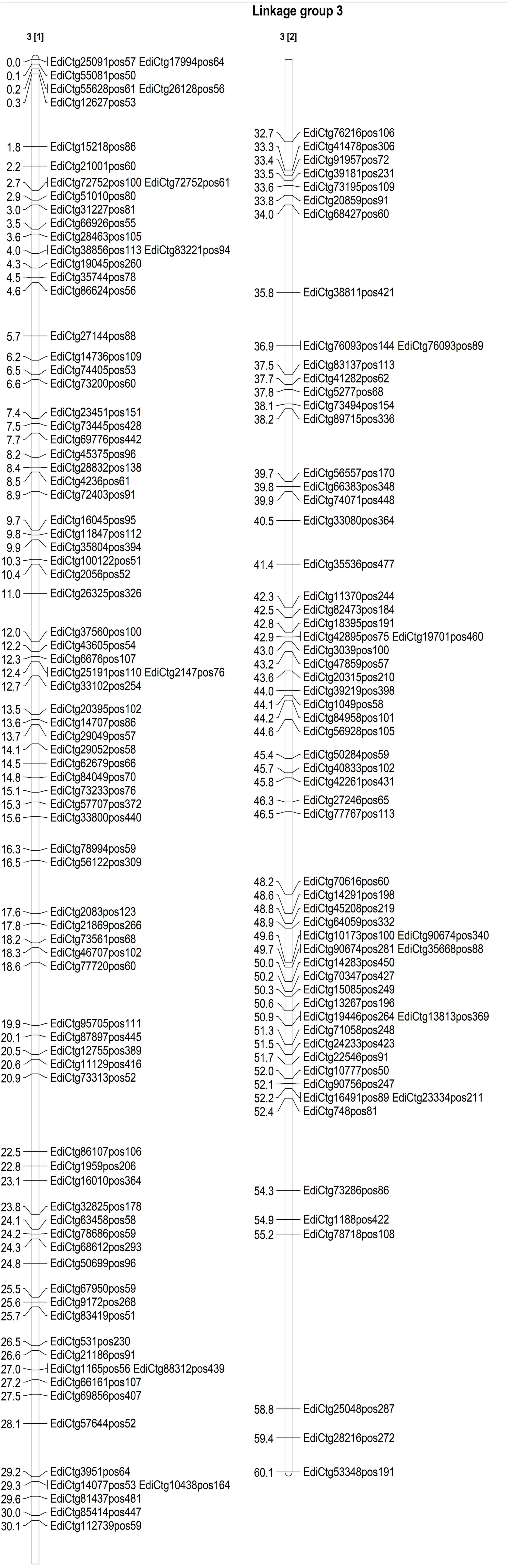

Linkage group 3
3 [1]
0.0 EdiCtg25091pos57 EdiCtg17994pos64
0.1 EdiCtg55081pos50
0.2 EdiCtg55628pos61 EdiCtg26128pos56
0.3 EdiCtg12627pos53
1.8 EdiCtg15218pos86
2.2 EdiCtg21001pos60
2.7 EdiCtg72752pos100 EdiCtg72752pos61
2.9 EdiCtg51010pos80
3.0 EdiCtg31227pos81
3.5 EdiCtg66926pos55
3.6 EdiCtg28463pos105
4.0 EdiCtg38856pos113 EdiCtg83221pos94
4.3 EdiCtg19045pos260
4.5 EdiCtg35744pos78
4.6 EdiCtg86624pos56
5.7 EdiCtg27144pos88
6.2 EdiCtg14736pos109
6.5 EdiCtg74405pos53
6.6 EdiCtg73200pos60
7.4 EdiCtg23451pos151
7.5 EdiCtg73445pos428
7.7 EdiCtg69776pos442
8.2 EdiCtg45375pos96
8.4 EdiCtg28832pos138
8.5 EdiCtg4236pos61
8.9 EdiCtg72403pos91
9.7 EdiCtg16045pos95
9.8 EdiCtg11847pos112
9.9 EdiCtg35804pos394
10.3 EdiCtg100122pos51
10.4 EdiCtg2056pos52
11.0 EdiCtg26325pos326
12.0 EdiCtg37560pos100
12.2 EdiCtg43605pos54
12.3 EdiCtg6676pos107
12.4 EdiCtg25191pos110 EdiCtg2147pos76
12.7 EdiCtg33102pos254
13.5 EdiCtg20395pos102
13.6 EdiCtg14707pos86
13.7 EdiCtg29049pos57
14.1 EdiCtg29052pos58
14.5 EdiCtg62679pos66
14.8 EdiCtg84049pos70
15.1 EdiCtg73233pos76
15.3 EdiCtg57707pos372
15.6 EdiCtg33800pos440
16.3 EdiCtg78994pos59
16.5 EdiCtg56122pos309
17.6 EdiCtg2083pos123
17.8 EdiCtg21869pos266
18.2 EdiCtg73561pos68
18.3 EdiCtg46707pos102
18.6 EdiCtg77720pos60
19.9 EdiCtg95705pos111
20.1 EdiCtg87897pos445
20.5 EdiCtg12755pos389
20.6 EdiCtg11129pos416
20.9 EdiCtg73313pos52
22.5 EdiCtg86107pos106
22.8 EdiCtg1959pos206
23.1 EdiCtg16010pos364
23.8 EdiCtg32825pos178
24.1 EdiCtg63458pos58
24.2 EdiCtg78686pos59
24.3 EdiCtg68612pos293
24.8 EdiCtg50699pos96
25.5 EdiCtg67950pos59
25.6 EdiCtg9172pos268
25.7 EdiCtg83419pos51
26.5 EdiCtg531pos230
26.6 EdiCtg21186pos91
27.0 EdiCtg1165pos56 EdiCtg88312pos439
27.2 EdiCtg66161pos107
27.5 EdiCtg69856pos407
28.1 EdiCtg57644pos52
29.2 EdiCtg3951pos64
29.3 EdiCtg14077pos53 EdiCtg10438pos164
29.6 EdiCtg81437pos481
30.0 EdiCtg85414pos447
30.1 EdiCtg112739pos59
3 [2]
32.7 EdiCtg76216pos106
33.3 EdiCtg41478pos306
33.4 EdiCtg91957pos72
33.5 EdiCtg39181pos231
33.6 EdiCtg73195pos109
33.8 EdiCtg20859pos91
34.0 EdiCtg68427pos60
35.8 EdiCtg38811pos421
36.9 EdiCtg76093pos144 EdiCtg76093pos89
37.5 EdiCtg83137pos113
37.7 EdiCtg41282pos62
37.8 EdiCtg5277pos68
38.1 EdiCtg73494pos154
38.2 EdiCtg89715pos336
39.7 EdiCtg56557pos170
39.8 EdiCtg66383pos348
39.9 EdiCtg74071pos448
40.5 EdiCtg33080pos364
41.4 EdiCtg35536pos477
42.3 EdiCtg11370pos244
42.5 EdiCtg82473pos184
42.8 EdiCtg18395pos191
42.9 EdiCtg42895pos75 EdiCtg19701pos460
43.0 EdiCtg3039pos100
43.2 EdiCtg47859pos57
43.6 EdiCtg20315pos210
44.0 EdiCtg39219pos398
44.1 EdiCtg1049pos58
44.2 EdiCtg84958pos101
44.6 EdiCtg56928pos105
45.4 EdiCtg50284pos59
45.7 EdiCtg40833pos102
45.8 EdiCtg42261pos431
46.3 EdiCtg27246pos65
46.5 EdiCtg77767pos113
48.2 EdiCtg70616pos60
48.6 EdiCtg14291pos198
48.8 EdiCtg45208pos219
48.9 EdiCtg64059pos332
49.6 EdiCtg10173pos100 EdiCtg90674pos340
49.7 EdiCtg90674pos281 EdiCtg35668pos88
50.0 EdiCtg14283pos450
50.2 EdiCtg70347pos427
50.3 EdiCtg15085pos249
50.6 EdiCtg13267pos196
50.9 EdiCtg19446pos264 EdiCtg13813pos369
51.3 EdiCtg71058pos248
51.5 EdiCtg24233pos423
51.7 EdiCtg22546pos91
52.0 EdiCtg10777pos50
52.1 EdiCtg90756pos247
52.2 EdiCtg16491pos89 EdiCtg23334pos211
52.4 EdiCtg748pos81
54.3 EdiCtg73286pos86
54.9 EdiCtg1188pos422
55.2 EdiCtg78718pos108
58.8 EdiCtg25048pos287
59.4 EdiCtg28216pos272
60.1 EdiCtg53348pos191

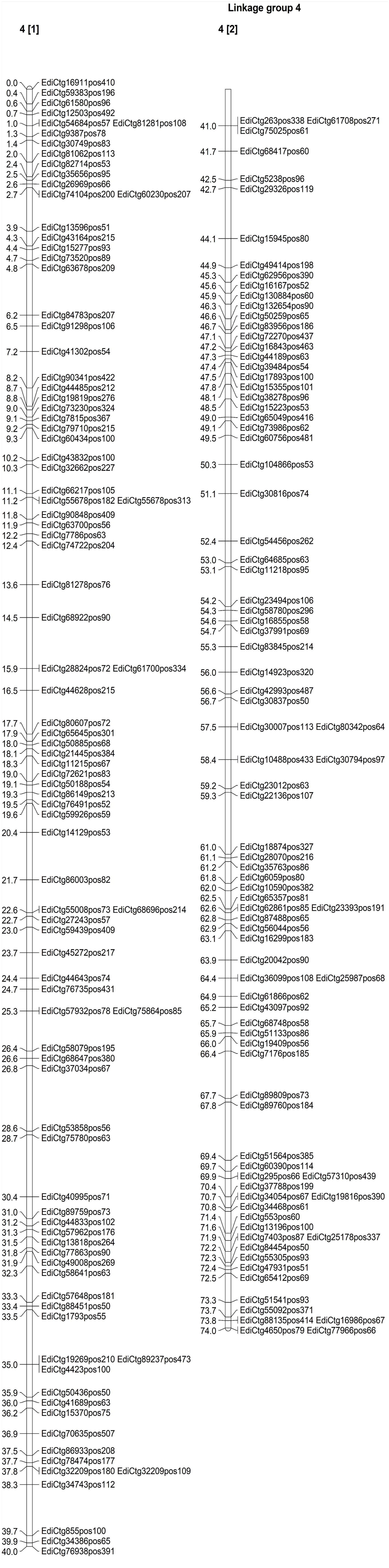
Linkage group 4
4 [1]
4 [2]
0.0 EdiCtg16911pos410
0.4 EdiCtg59383pos196
0.6 EdiCtg61580pos96
0.7 EdiCtg12503pos492
1.0 EdiCtg54684pos57 EdiCtg81281pos108
1.3 EdiCtg9387pos78
1.4 EdiCtg30749pos83
2.0 EdiCtg81062pos113
2.4 EdiCtg82714pos53
2.5 EdiCtg35656pos95
2.6 EdiCtg26969pos66
2.7 EdiCtg74104pos200 EdiCtg60230pos207
3.9 EdiCtg13596pos51
4.3 EdiCtg43164pos215
4.4 EdiCtg15277pos93
4.7 EdiCtg73520pos89
4.8 EdiCtg63678pos209
6.2 EdiCtg84783pos207
6.5 EdiCtg91298pos106
7.2 EdiCtg41302pos54
8.2 EdiCtg90341pos422
8.7 EdiCtg44485pos212
8.8 EdiCtg19819pos276
9.0 EdiCtg73230pos324
9.1 EdiCtg7815pos367
9.2 EdiCtg79710pos215
9.3 EdiCtg60434pos100
10.2 EdiCtg43832pos100
10.3 EdiCtg32662pos227
11.1 EdiCtg66217pos105
11.2 EdiCtg55678pos182 EdiCtg55678pos313
11.8 EdiCtg90848pos409
11.9 EdiCtg63700pos56
12.2 EdiCtg7786pos63
12.4 EdiCtg74722pos204
13.6 EdiCtg81278pos76
14.5 EdiCtg68922pos90
15.9 EdiCtg28824pos72 EdiCtg61700pos334
16.5 EdiCtg44628pos215
17.7 EdiCtg80607pos72
17.9 EdiCtg65645pos301
18.0 EdiCtg50885pos68
18.1 EdiCtg21445pos384
18.3 EdiCtg11215pos67
19.0 EdiCtg72621pos83
19.1 EdiCtg50188pos54
19.3 EdiCtg86149pos213
19.5 EdiCtg76491pos52
19.6 EdiCtg59926pos59
20.4 EdiCtg14129pos53
21.7 EdiCtg86003pos82
22.6 EdiCtg55008pos73 EdiCtg68696pos214
22.7 EdiCtg27243pos57
23.0 EdiCtg59439pos409
23.7 EdiCtg45272pos217
24.4 EdiCtg44643pos74
24.7 EdiCtg76735pos431
25.3 EdiCtg57932pos78 EdiCtg75864pos85
26.4 EdiCtg58079pos195
26.6 EdiCtg68647pos380
26.8 EdiCtg37034pos67
28.6 EdiCtg53858pos56
28.7 EdiCtg75780pos63
30.4 EdiCtg40995pos71
31.0 EdiCtg89759pos73
31.2 EdiCtg44833pos102
31.3 EdiCtg57962pos176
31.5 EdiCtg13818pos264
31.8 EdiCtg77863pos90
31.9 EdiCtg49008pos269
32.3 EdiCtg58641pos63
33.3 EdiCtg57648pos181
33.4 EdiCtg88451pos50
33.5 EdiCtg1793pos55
35.0 EdiCtg19269pos210 EdiCtg89237pos473
EdiCtg4423pos100
35.9 EdiCtg50436pos50
36.0 EdiCtg41689pos63
36.2 EdiCtg15370pos75
36.9 EdiCtg70635pos507
37.5 EdiCtg86933pos208
37.7 EdiCtg78474pos177
37.8 EdiCtg32209pos180 EdiCtg32209pos109
38.3 EdiCtg34743pos112
39.7 EdiCtg855pos100
39.9 EdiCtg34386pos65
40.0 EdiCtg76938pos391
41.0 EdiCtg263pos338 EdiCtg61708pos271
EdiCtg75025pos61
41.7 EdiCtg68417pos60
42.5 EdiCtg5238pos96
42.7 EdiCtg29326pos119
44.1 EdiCtg15945pos80
44.9 EdiCtg49414pos198
45.3 EdiCtg62956pos390
45.6 EdiCtg16167pos52
45.9 EdiCtg130884pos60
46.3 EdiCtg132654pos90
46.6 EdiCtg50259pos65
46.7 EdiCtg83956pos186
47.1 EdiCtg72270pos437
47.2 EdiCtg16843pos463
47.3 EdiCtg44189pos63
47.4 EdiCtg39484pos54
47.5 EdiCtg17893pos100
47.8 EdiCtg15355pos101
48.1 EdiCtg38278pos96
48.5 EdiCtg15223pos53
49.0 EdiCtg65049pos416
49.1 EdiCtg73986pos62
49.5 EdiCtg60756pos481
50.3 EdiCtg104866pos53
51.1 EdiCtg30816pos74
52.4 EdiCtg54456pos262
53.0 EdiCtg64685pos63
53.1 EdiCtg11218pos95
54.2 EdiCtg23494pos106
54.3 EdiCtg58780pos296
54.6 EdiCtg16855pos58
54.7 EdiCtg37991pos69
55.3 EdiCtg83845pos214
56.0 EdiCtg14923pos320
56.6 EdiCtg42993pos487
56.7 EdiCtg30837pos50
57.5 EdiCtg30007pos113 EdiCtg80342pos64
58.4 EdiCtg10488pos433 EdiCtg30794pos97
59.2 EdiCtg23012pos63
59.3 EdiCtg22136pos107
61.0 EdiCtg18874pos327
61.1 EdiCtg28070pos216
61.2 EdiCtg35763pos86
61.8 EdiCtg6059pos80
62.0 EdiCtg10590pos382
62.5 EdiCtg65357pos81
62.6 EdiCtg62861pos85 EdiCtg23393pos191
62.8 EdiCtg87488pos65
62.9 EdiCtg56044pos56
63.1 EdiCtg16299pos183
63.9 EdiCtg20042pos90
64.4 EdiCtg36099pos108 EdiCtg25987pos68
64.9 EdiCtg61866pos62
65.2 EdiCtg43097pos92
65.7 EdiCtg68748pos58
65.9 EdiCtg51133pos86
66.0 EdiCtg19409pos56
66.4 EdiCtg7176pos185
67.7 EdiCtg89809pos73
67.8 EdiCtg89760pos184
69.4 EdiCtg51564pos385
69.7 EdiCtg60390pos114
69.9 EdiCtg295pos66 EdiCtg57310pos439
70.4 EdiCtg37788pos199
70.7 EdiCtg34054pos67 EdiCtg19816pos390
70.8 EdiCtg34468pos61
71.4 EdiCtg553pos60
71.6 EdiCtg13196pos100
71.9 EdiCtg7403pos87 EdiCtg25178pos337
72.2 EdiCtg84454pos50
72.3 EdiCtg55305pos93
72.4 EdiCtg47931pos51
72.5 EdiCtg65412pos69
73.3 EdiCtg51541pos93
73.7 EdiCtg55092pos371
73.8 EdiCtg88135pos414 EdiCtg16986pos67
74.0 EdiCtg4650pos79 EdiCtg77966pos66

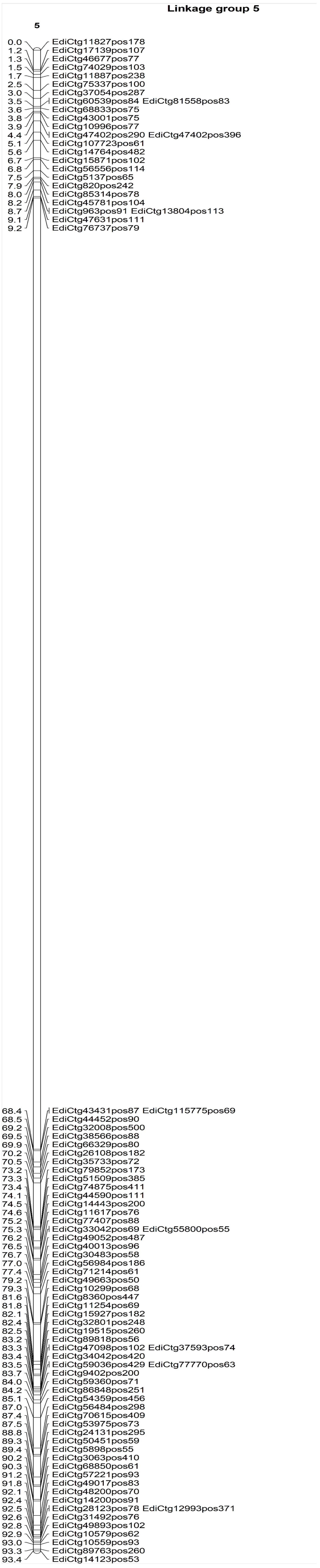
Linkage group 5
5
0.0 EdiCtg11827pos178
1.2 EdiCtg17139pos107
1.3 EdiCtg46677pos77
1.5 EdiCtg74029pos103
1.7 EdiCtg11887pos238
2.5 EdiCtg75337pos100
3.0 EdiCtg37054pos287
3.5 EdiCtg60539pos84 EdiCtg81558pos83
3.6 EdiCtg68833pos75
3.8 EdiCtg43001pos75
3.9 EdiCtg10996pos77
4.4 EdiCtg47402pos290 EdiCtg47402pos396
5.1 EdiCtg107723pos61
5.6 EdiCtg14764pos482
6.7 EdiCtg15871pos102
6.8 EdiCtg56556pos114
7.5 EdiCtg5137pos65
7.9 EdiCtg820pos242
8.0 EdiCtg85314pos78
8.2 EdiCtg45781pos104
8.7 EdiCtg963pos91 EdiCtg13804pos113
9.1 EdiCtg47631pos111
9.2 EdiCtg76737pos79
68.4 EdiCtg43431pos87 EdiCtg115775pos69
68.5 EdiCtg44452pos90
69.2 EdiCtg32008pos500
69.5 EdiCtg38566pos88
69.9 EdiCtg66329pos80
70.2 EdiCtg26108pos182
70.5 EdiCtg35733pos72
73.2 EdiCtg79852pos173
73.3 EdiCtg51509pos385
73.4 EdiCtg74875pos411
74.1 EdiCtg44590pos111
74.5 EdiCtg14443pos200
74.6 EdiCtg11617pos76
75.2 EdiCtg77407pos88
75.3 EdiCtg33042pos69 EdiCtg55800pos55
76.2 EdiCtg49052pos487
76.5 EdiCtg40013pos96
76.7 EdiCtg30483pos58
77.0 EdiCtg56984pos186
77.4 EdiCtg71214pos61
79.2 EdiCtg49663pos50
79.3 EdiCtg10299pos68
81.6 EdiCtg8360pos447
81.8 EdiCtg11254pos69
82.1 EdiCtg15927pos182
82.4 EdiCtg32801pos248
82.5 EdiCtg19515pos260
83.2 EdiCtg89818pos56
83.3 EdiCtg47098pos102 EdiCtg37593pos74
83.4 EdiCtg34042pos420
83.5 EdiCtg59036pos429 EdiCtg77770pos63
83.7 EdiCtg9402pos200
84.0 EdiCtg59360pos71
84.2 EdiCtg86848pos251
85.1 EdiCtg54359pos456
87.0 EdiCtg56484pos298
87.4 EdiCtg70615pos409
87.5 EdiCtg53975pos73
88.8 EdiCtg24131pos295
89.3 EdiCtg50451pos59
89.4 EdiCtg5898pos55
90.2 EdiCtg3063pos410
90.3 EdiCtg68850pos61
91.2 EdiCtg57221pos93
91.8 EdiCtg49017pos83
92.1 EdiCtg48200pos70
92.4 EdiCtg14200pos91
92.5 EdiCtg28123pos78 EdiCtg12993pos371
92.6 EdiCtg31492pos76
92.8 EdiCtg49893pos102
92.9 EdiCtg10579pos62
93.0 EdiCtg10559pos93
93.3 EdiCtg89763pos260
93.4 EdiCtg14123pos53

Linkage group 6

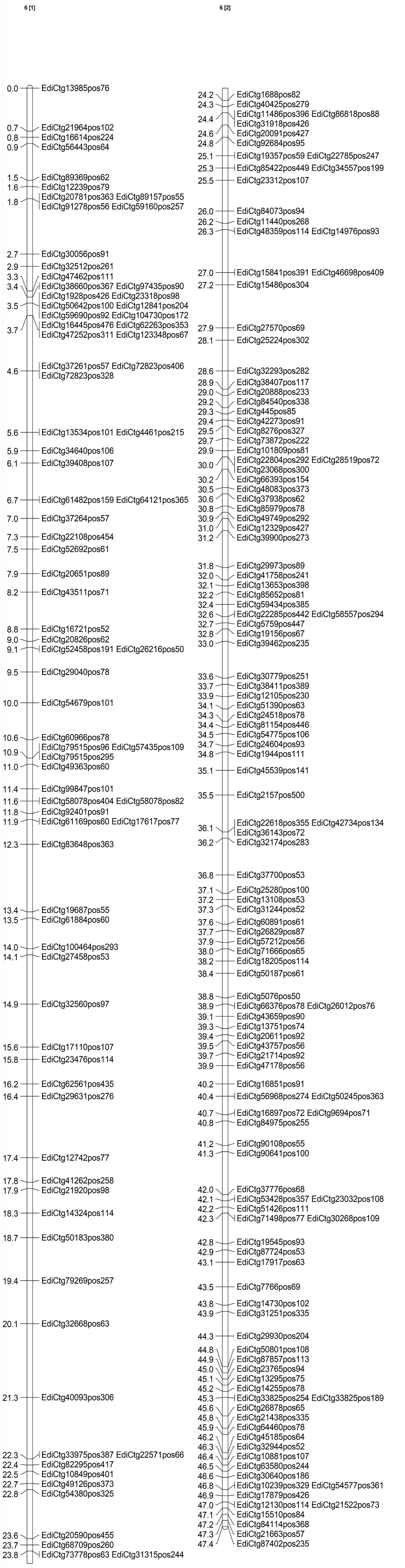

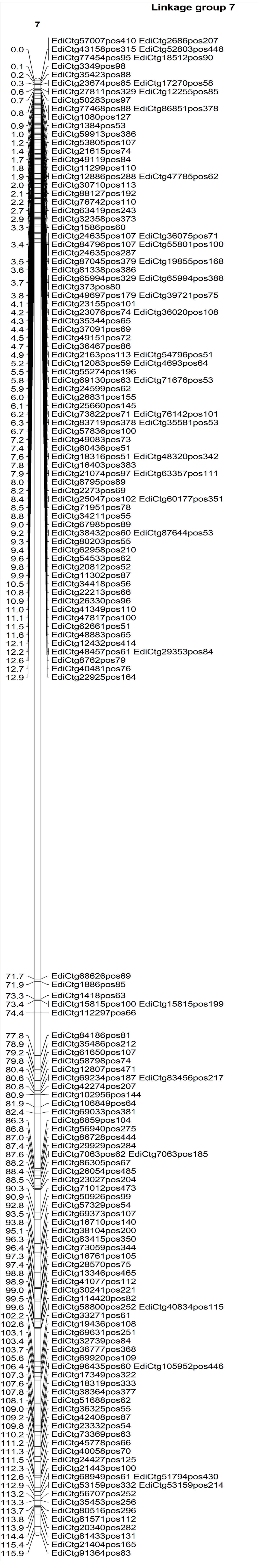
Linkage group 7
7
0.0
0.1
0.2
0.3
0.6
0.7
0.8
0.9
1.0
1.2
1.4
1.7
1.8
1.9
2.0
2.1
2.2
2.7
2.9
3.3
3.4
3.5
3.6
3.7
3.8
4.1
4.2
4.3
4.4
4.5
4.7
4.9
5.2
5.5
5.8
5.9
6.0
6.1
6.2
6.3
6.7
7.2
7.4
7.6
7.8
7.9
8.0
8.2
8.4
8.5
8.8
9.0
9.2
9.3
9.4
9.6
9.8
9.9
10.5
10.8
10.9
11.0
11.1
11.5
11.6
12.1
12.2
12.6
12.7
12.9
71.7
71.9
73.3
73.4
74.4
77.8
78.9
79.2
79.8
80.4
80.6
80.8
80.9
81.9
82.4
86.3
86.8
87.0
87.4
87.6
88.2
88.4
88.5
90.3
90.9
92.8
93.5
93.8
95.1
96.3
96.4
97.3
97.4
98.8
98.9
99.0
99.5
99.6
102.2
102.6
103.1
103.4
103.7
105.6
106.4
107.3
107.6
107.8
108.1
109.0
109.2
109.8
110.2
111.2
111.3
111.5
112.3
112.6
112.9
113.2
113.3
113.7
113.8
113.9
114.4
115.4
115.9
EdiCtg57007pos410 EdiCtg2686pos207
EdiCtg43158pos315 EdiCtg52803pos448
EdiCtg77454pos95 EdiCtg18512pos90
EdiCtg3349pos98
EdiCtg35423pos88
EdiCtg23674pos85 EdiCtg17270pos58
EdiCtg27811pos329 EdiCtg12255pos85
EdiCtg50283pos97
EdiCtg77468pos88 EdiCtg86851pos378
EdiCtg1080pos127
EdiCtg1384pos53
EdiCtg59913pos386
EdiCtg53805pos107
EdiCtg21615pos74
EdiCtg49119pos84
EdiCtg11299pos110
EdiCtg12886pos288 EdiCtg47785pos62
EdiCtg30710pos113
EdiCtg88127pos192
EdiCtg76742pos110
EdiCtg63419pos243
EdiCtg32358pos373
EdiCtg1586pos60
EdiCtg24635pos107 EdiCtg36075pos71
EdiCtg84796pos107 EdiCtg55801pos100
EdiCtg24635pos287
EdiCtg87045pos379 EdiCtg19855pos168
EdiCtg81338pos386
EdiCtg65994pos329 EdiCtg65994pos388
EdiCtg373pos80
EdiCtg49697pos179 EdiCtg39721pos75
EdiCtg23155pos101
EdiCtg23076pos74 EdiCtg36020pos108
EdiCtg35344pos65
EdiCtg37091pos69
EdiCtg49151pos72
EdiCtg36467pos86
EdiCtg2163pos113 EdiCtg54796pos51
EdiCtg12083pos59 EdiCtg4693pos64
EdiCtg55274pos196
EdiCtg69130pos63 EdiCtg71676pos53
EdiCtg24599pos62
EdiCtg26831pos155
EdiCtg25660pos145
EdiCtg73822pos71 EdiCtg76142pos101
EdiCtg83719pos378 EdiCtg35581pos53
EdiCtg57836pos100
EdiCtg49083pos73
EdiCtg60436pos51
EdiCtg18316pos51 EdiCtg48320pos342
EdiCtg16403pos383
EdiCtg21074pos97 EdiCtg63357pos111
EdiCtg8795pos89
EdiCtg2273pos69
EdiCtg25047pos102 EdiCtg60177pos351
EdiCtg71951pos78
EdiCtg34211pos55
EdiCtg67985pos89
EdiCtg38432pos60 EdiCtg87644pos53
EdiCtg80203pos55
EdiCtg62958pos210
EdiCtg54533pos62
EdiCtg20812pos52
EdiCtg11302pos87
EdiCtg34418pos56
EdiCtg22213pos66
EdiCtg26330pos96
EdiCtg41349pos110
EdiCtg47817pos100
EdiCtg62661pos51
EdiCtg48883pos65
EdiCtg12432pos414
EdiCtg48457pos61 EdiCtg29353pos84
EdiCtg8762pos79
EdiCtg40481pos76
EdiCtg22925pos164
EdiCtg68626pos69
EdiCtg1886pos85
EdiCtg1418pos63
EdiCtg15815pos100 EdiCtg15815pos199
EdiCtg112297pos66
EdiCtg84186pos81
EdiCtg35486pos212
EdiCtg61650pos107
EdiCtg58798pos74
EdiCtg12807pos471
EdiCtg69234pos187 EdiCtg83456pos217
EdiCtg42274pos207
EdiCtg102956pos144
EdiCtg106849pos64
EdiCtg69033pos381
EdiCtg8859pos104
EdiCtg56940pos275
EdiCtg86728pos444
EdiCtg29929pos284
EdiCtg7063pos62 EdiCtg7063pos185
EdiCtg86305pos67
EdiCtg26054pos485
EdiCtg23027pos204
EdiCtg71012pos473
EdiCtg50926pos99
EdiCtg57329pos54
EdiCtg69373pos107
EdiCtg16710pos140
EdiCtg38104pos200
EdiCtg83415pos350
EdiCtg73059pos344
EdiCtg16761pos105
EdiCtg28570pos75
EdiCtg13346pos465
EdiCtg41077pos112
EdiCtg30241pos221
EdiCtg114420pos82
EdiCtg58800pos252 EdiCtg40834pos115
EdiCtg33271pos61
EdiCtg19436pos108
EdiCtg69631pos251
EdiCtg32739pos84
EdiCtg36777pos368
EdiCtg69920pos109
EdiCtg96435pos60 EdiCtg105952pos446
EdiCtg17349pos322
EdiCtg18319pos333
EdiCtg38364pos377
EdiCtg51688pos62
EdiCtg36325pos55
EdiCtg42408pos87
EdiCtg23332pos54
EdiCtg73369pos63
EdiCtg45778pos66
EdiCtg40058pos70
EdiCtg24427pos125
EdiCtg21443pos100
EdiCtg68949pos61 EdiCtg51794pos430
EdiCtg53159pos332 EdiCtg53159pos214
EdiCtg56707pos252
EdiCtg35453pos256
EdiCtg80516pos296
EdiCtg81571pos112
EdiCtg20340pos282
EdiCtg81433pos131
EdiCtg21404pos165
EdiCtg91364pos83

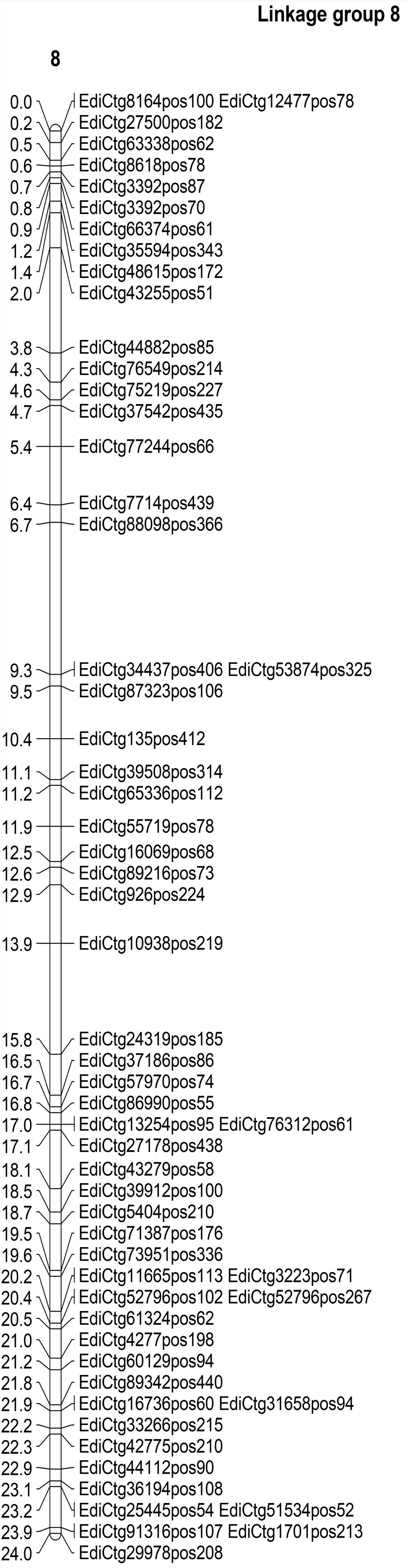
Linkage group 8
8
0.0 EdiCtg8164pos100 EdiCtg12477pos78
0.2 EdiCtg27500pos182
0.5 EdiCtg63338pos62
0.6 EdiCtg8618pos78
0.7 EdiCtg3392pos87
0.8 EdiCtg3392pos70
0.9 EdiCtg66374pos61
1.2 EdiCtg35594pos343
1.4 EdiCtg48615pos172
2.0 EdiCtg43255pos51
3.8 EdiCtg44882pos85
4.3 EdiCtg76549pos214
4.6 EdiCtg75219pos227
4.7 EdiCtg37542pos435
5.4 EdiCtg77244pos66
6.4 EdiCtg7714pos439
6.7 EdiCtg88098pos366
9.3 EdiCtg34437pos406 EdiCtg53874pos325
9.5 EdiCtg87323pos106
10.4 EdiCtg135pos412
11.1 EdiCtg39508pos314
11.2 EdiCtg65336pos112
11.9 EdiCtg55719pos78
12.5 EdiCtg16069pos68
12.6 EdiCtg89216pos73
12.9 EdiCtg926pos224
13.9 EdiCtg10938pos219
15.8 EdiCtg24319pos185
16.5 EdiCtg37186pos86
16.7 EdiCtg57970pos74
16.8 EdiCtg86990pos55
17.0 EdiCtg13254pos95 EdiCtg76312pos61
17.1 EdiCtg27178pos438
18.1 EdiCtg43279pos58
18.5 EdiCtg39912pos100
18.7 EdiCtg5404pos210
19.5 EdiCtg71387pos176
19.6 EdiCtg73951pos336
20.2 EdiCtg11665pos113 EdiCtg3223pos71
20.4 EdiCtg52796pos102 EdiCtg52796pos267
20.5 EdiCtg61324pos62
21.0 EdiCtg4277pos198
21.2 EdiCtg60129pos94
21.8 EdiCtg89342pos440
21.9 EdiCtg16736pos60 EdiCtg31658pos94
22.2 EdiCtg33266pos215
22.3 EdiCtg42775pos210
22.9 EdiCtg44112pos90
23.1 EdiCtg36194pos108
23.2 EdiCtg25445pos54 EdiCtg51534pos52
23.9 EdiCtg91316pos107 EdiCtg1701pos213
24.0 EdiCtg29978pos208

Supplentary Table 1. DNA contents (pg/2C) of accessions or populations of *Lepidium campestre* and *Lepidium heterophyllum*

Lepidium ID	Species	Source	Provenance	Country	Genome size
PI 633250	*L. campestre*	USDA-ARS[a]	Leipzig	Germany	0.406
PI 633252	*L. campestre*	USDA-ARS	Molsheim	France	0.412
PI 633249	*L. campestre*	USDA-ARS	Uhrovec	Slovak	0.433
PI 633248	*L. campestre*	USDA-ARS	Ruderal	Czech	0.412
Ljugarn	*L. campestre*	Collection	Gotland	Sweden	0.412
Höör-1	*L. campestre*	Collection	Skåne	Sweden	0.408
Höör-2	*L. campestre*	Collection	Skåne	Sweden	0.410
Albrunna	*L. campestre*	Collection	Öland	Sweden	0.412
Gävle	*L. campestre*	Collection	Gävleborg	Sweden	0.416
Skövde	*L. campestre*	Collection	VästerGöteland	Sweden	0.394
Risekatslösa	*L. campestre*	Collection	Skåne	Sweden	0.425
Skoldrebacken	*L. campestre*	Collection	Gotland	Sweden	0.421
Örebro	*L. campestre*	Collection	Närke	Sweden	0.415
Viken	*L. campestre*	Collection	Skåne	Sweden	0.423
Västerås-1	*L. campestre*	Collection	Västmansland	Sweden	0.413
Västerås-2	*L. campestre*	Collection	Västmansland	Sweden	0.411
Uppsala-1	*L. campestre*	Collection	Uppland	Sweden	0.418
Uppsala-2	*L. campestre*	Collection	Uppland	Sweden	0.418
Trelleborg	*L. campestre*	Collection	Skåne	Sweden	0.427
Spjutstrop	*L. campestre*	Collection	Skåne	Sweden	0.412
Stuvsta	*L. campestre*	Collection	Södermanland	Sweden	0.425
Ventlinge	*L. campestre*	Collection	Öland	Sweden	0.435
Norra Vram	*L. campestre*	Collection	Skåne	Sweden	0.421
Näsudden	*L. campestre*	Collection	Gotland	Sweden	0.414
Mörbylånga	*L. campestre*	Collection	Öland	Sweden	0.439
Arrie	*L. campestre*	Collection	Skåne	Sweden	0.430
Lep 94	*L. campestre*	IPK[b]	Unknown	Germany	0.406
Kalkstad	*L. campestre*	Collection	Öland	Sweden	0.433
NGB22634	*L. campestre*	NordGen gene bank	Unknown	Denmark	0.399
Lep 122	*L. campestre*	IPK	Eisenach	Germany	0.406
Lep 91	*L. campestre*	IPK	Unknown	Greece	0.423
Lep 89	*L. campestre*	IPK	Makedonien	Greece	0.430
Lep 93	*L. campestre*	IPK	Gimritz bei Wettin	Germany	0.406
Huddinge	*L. campestre*	Collection	Stockholm	Sweden	0.414
Lep 92	*L. campestre*	IPK	Unknown	Greece	0.408
Lep 124	*L. campestre*	IPK	Unknown	France	0.399
PI 597853	*L. campestre*	USDA-ARS	Leon	Spain	0.414
PI 633251	*L. campestre*	USDA-ARS	Leipzig-Connewitz	Germany	0.403
PI 650259	*L. campestre*	USDA-ARS	Reichenbach	Germany	0.415
PI 650260	*L. campestre*	USDA-ARS	Leipzig-Mockau	Germany	0.405
Lönstrap	*L. campestre*	Unknown	unknown	Unknown	0.429
Malmö	*L. campestre*	Collection	Skåne	Sweden	0.419
Grönhögen	*L. campestre*	Collection	Öland	Sweden	0.429
Årsta	*L. campestre*	Collection	Södermanland	Sweden	0.409
Kristianstad	*L. campestre*	Collection	Skåne	Sweden	0.417
0018580	*L. campestre*	Millenium Seed Bank Project, West Sussex	Unknown	United Kingdom	0.402
LC_EHA_205/phyl(2X)-1A	*L. campestre*	Unknown	Unknown	Unknown	0.415
49324	*L. campestre*	Unknown	Unknown	Unknown	0.409
				Mean	0.416
				SD	0.010
PI 597856	*L. heterophyllum*	USDA-ARS	Sierra Guadarrama	Spain	0.409
PI 597856-3B	*L. heterophyllum*	USDA-ARS	Sierra Guadarrama	Spain	0.403
Hästvada	*L. heterophyllum*	New collection	Skåne	Sweden	0.410
				Mean	0.407
				SD	0.003

[a]United States Department of Agriculture - Agricultural Research Service, [b]Genebank Leibniz Institute of Plant Genetics and Crop Plant Research, Gatersleben, Germany

Supplementary Table 2. Name of accessions and hybrids (Hys) of *Lepidium* used for molecular cytogenetic analysis

Lepidium ID	Sample code	*Species*	Remark
NO94-6	BSt_2	*L. campestre*	
Lep 124	G45_1	*L. campestre*	
LC_EHA_205/ phyl (2X)-1A	C54_1	*L. campestre*	
PI 597856	BP1_1	*L. heterophyllum*	
Hästvada	C66_4	*L. heterophyllum*	
Hy25_146_1	Hy25_146	Hybrid of *L campestre* X *L. heterophyllum*	F_3 hybrid
Hy25_23_1	Hy56_1	Hybrid of *L campestre* X *L. heterophyllum*	F_4 hybrid
Hy25_279_1	Hy58	Hybrid of *L campestre* X *L. heterophyllum*	F_4 hybrid

Supplementary Table 5. Duplicated loci in *Lepidium campestre* in comparison with *Arabidopsis* genome

SNP_ID	Linkage group (LG)	Genetic distance	Chromosome	Duplicated *Arabidopsis* gene	E-value	Identity
EdiCtg34741pos431	LG1	0.208	Chromosome 4	AT4G00460	0	89%
EdiCtg34741pos86	LG1	0.208	Chromosome 4	AT4G00460	0	89%
EdiCtg90334pos457	LG1	1.143	Chromosome 4	AT4G00990	1E-137	85%
EdiCtg90334pos73	LG1	1.143	Chromosome 4	AT4G00990	1E-137	85%
EdiCtg61394pos59	LG1	4.989	Chromosome 4	AT4G02760	8E-26	79%
EdiCtg61394pos405	LG1	4.989	Chromosome 4	AT4G02760	2E-27	80%
EdiCtg14783pos357	LG1	10.068	Chromosome 4	AT4G04970	6E-171	88%
EdiCtg14783pos51	LG1	10.184	Chromosome 4	AT4G04970	6E-171	88%
EdiCtg10064pos368	LG1	23.106	Chromosome 4	AT4G09890	2E-52	83%
EdiCtg10064pos432	LG1	23.106	Chromosome 4	AT4G09890	2E-52	83%
EdiCtg16994pos232	LG1	23.418	Chromosome 4	AT4G09680	8E-125	82%
EdiCtg16994pos467	LG1	23.418	Chromosome 4	AT4G09680	8E-125	82%
EdiCtg54446pos257	LG1	40.925	Chromosome 5	AT5G52780	3E-104	88%
EdiCtg54446pos90	LG1	40.925	Chromosome 5	AT5G52780	3E-104	88%
EdiCtg56656pos347	LG1	41.967	Chromosome 5	AT5G53110	2E-12	92%
EdiCtg56656pos273	LG1	41.967	Chromosome 5	AT5G53110	2E-12	92%
EdiCtg58692pos436	LG1	59.066	Chromosome 5	AT5G57785	6E-62	88%
EdiCtg58692pos110	LG1	59.066	Chromosome 5	AT5G57785	3E-60	87%
EdiCtg57377pos244	LG1	67.075	Chromosome 5	AT5G20510	4E-163	86%
EdiCtg57377pos345	LG1	67.075	Chromosome 5	AT5G20510	4E-163	86%
EdiCtg42277pos485	LG1	69.986	Chromosome 5	AT5G64170	5E-97	86%
EdiCtg42277pos401	LG1	69.986	Chromosome 5	AT5G64170	1E-98	87%
EdiCtg21041pos471	LG2	36.752	Chromosome 5	AT5G23810	2E-107	82%
EdiCtg21041pos352	LG2	36.752	Chromosome 5	AT5G23810	2E-107	82%
EdiCtg84707pos468	LG2	66.042	Chromosome 5	AT5G44390	2E-115	82%
EdiCtg84707pos112	LG2	66.042	Chromosome 5	AT5G44390	1E-113	82%
EdiCtg72752pos61	LG3	2.723	Chromosome 1	AT1G02530	6E-114	91%
EdiCtg72752pos100	LG3	2.723	Chromosome 1	AT1G02520	2E-69	84%
EdiCtg90674pos340	LG3	49.625	Chromosome 1	AT1G48910	6E-37	84%
EdiCtg90674pos281	LG3	49.671	Chromosome 1	AT1G48910	6E-37	84%
EdiCtg76093pos144	LG3	36.866	Chromosome 5	AT5G43690	2E-18	85%
EdiCtg76093pos89	LG3	36.866	Chromosome 5	AT5G43690	2E-18	85%
EdiCtg32209pos180	LG4	37.779	Chromosome 1	AT1G42430	2E-72	82%
EdiCtg32209pos109	LG4	37.779	Chromosome 1	AT1G42430	2E-72	82%
EdiCtg79515pos96	LG6	10.857	Chromosome 1	AT1G66400	2E-56	79%
EdiCtg79515pos295	LG6	10.867	Chromosome 1	AT1G66400	2E-56	79%
EdiCtg58078pos404	LG6	11.574	Chromosome 1	AT1G66810	6E-57	78%
EdiCtg58078pos82	LG6	11.586	Chromosome 1	AT1G66810	6E-57	78%
EdiCtg24635pos107	LG7	3.432	Chromosome 2	AT2G43945	1E-57	77%
EdiCtg24635pos287	LG7	3.432	Chromosome 2	AT2G43945	3E-59	77%
EdiCtg65994pos388	LG7	3.744	Chromosome 2	AT2G43700	2E-131	82%
EdiCtg65994pos329	LG7	3.744	Chromosome 2	AT2G43700	4E-133	82%
EdiCtg53159pos332	LG7	112.944	Chromosome 2	AT2G21540	5E-23	82%
EdiCtg53159pos214	LG7	112.944	Chromosome 2	AT2G21540	5E-23	82%
EdiCtg52796pos267	LG8	20.352	Chromosome 4	AT4G32520	4E-173	88%
EdiCtg52796pos102	LG8	20.352	Chromosome 4	AT4G32520	4E-173	88%
